# The hyperfunction theory: an emerging paradigm for the biology of aging

**DOI:** 10.1016/j.arr.2021.101557

**Published:** 2022-01-03

**Authors:** David Gems

**Affiliations:** Institute of Healthy Ageing, and Research Department of Genetics, Evolution and Environment, University College London, London WC1E 6BT, UK

**Keywords:** antagonistic pleiotropy, insulin/IGF-1 signalling, hyperfunction, quasi-programs, mTOR, theories of aging, programmatic aging

## Abstract

The process of senescence (aging) is predominantly determined by the action of wild-type genes. For most organisms, this does not reflect any adaptive function that senescence serves, but rather evolutionary effects of declining selection against genes with deleterious effects later in life. To understand aging requires an account of how evolutionary mechanisms give rise to pathogenic gene action and late-life disease, that integrates evolutionary (ultimate) and mechanistic (proximate) causes into a single explanation. A well-supported evolutionary explanation by G.C. Williams argues that senescence can evolve due to pleiotropic effects of alleles with antagonistic effects on fitness and late-life health (antagonistic pleiotropy, AP). What has remained unclear is how gene action gives rise to late-life disease pathophysiology. One ultimate-proximate account is T.B.L. Kirkwood’s disposable soma theory. Based on the hypothesis that stochastic molecular damage causes senescence, this reasons that aging is coupled to reproductive fitness due to preferential investment of resources into reproduction, rather than somatic maintenance. An alternative and more recent ultimate-proximate theory argues that aging is largely caused by programmatic, developmental-type mechanisms. Here ideas about AP and programmatic aging are reviewed, particularly those of M.V. Blagosklonny (the hyperfunction theory) and J.P. de Magalhães (the developmental theory), and their capacity to make sense of diverse experimental findings is assessed.

## 1 Introduction

As a field, the biology of senescence (aging) is lacking in terms of a core explanatory framework or paradigm such as that possessed by chemistry or genetics (Gems and de Magalhães, 2021). This review surveys an emerging set of ideas which for convenience will be referred to here as the programmatic theory. These ideas offer a new account of the proximate mechanisms of antagonistic pleiotropy, an important evolutionary-genetic principle in the biology of aging (Williams, 1957).

Devising effective explanations of senescence as a whole is a challenge that has, unfortunately, defeated biogerontologists. Why is aging so difficult to understand? Arguably, there are two reasons in particular. First, because it is a multifactorial process, as reflected in the fact that diseases of aging are largely multifactorial in terms of their etiology. Thus, any individual theory about a given senescent etiology will be insufficient to explain the whole phenomenon. Second, because the relative contribution of the factors that together cause aging is variable and context dependent, differing according to the senescent pathology considered, to environmental conditions and type of organism (Ezcurra et al., 2018; Gems and de Magalhães, 2021).

Though this presents a difficult challenge, it does not mean that senescence is impossible to understand. Though aging is a multifactorial process, a plausible hope is that the number of individual factors involved is relatively small, particularly when viewed in terms of broad determinative principles. Moreover, given the common evolutionary origins of animal species, these principles are likely to be operative in diverse organisms. Thus, the challenge for biogerontology is to understand these individual principles, and how they combine to cause aging-related disease, and aging as a whole (Gems and de Magalhães, 2021).

While attempts to understand aging from the 1990s onwards were strongly focused on the role of accumulation of molecular damage as a cause of aging, earlier accounts tended to be more broadly multifactorial (Comfort, 1979; Cutler, 1984; Dilman, 1994; Finch, 1990; Strehler, 1977). Multifactorial models, particularly that of Vladimir Dilman, will be discussed later in this article. In the meantime, it should be emphasized that programmatic mechanisms and antagonistic pleiotropy (AP) as described here are elements of a wider, multifactorial process.

In the following discussion I will first introduce the concept of AP, as described by George Williams, and then go on to describe attempts to explain AP in terms of actual biological mechanisms (e.g. biochemical, cellular, developmental) that form a pathophysiological basis of the process of senescence. These include Tom Kirkwood’s disposable soma theory, Misha Blagosklonny’s hyperfunction theory, João Pedro de Magalhães’s developmental theory, and Vladimir Dilman’s ontogenetic theory. The latter three theories share key features, and can be referred to collectively as the programmatic theory. For definitions of key terms, see the glossary.

## 2 The evolution of aging

Diseases of aging are strange in that their principal etiology is not any of the usual suspects that disrupt normal function (infectious pathogens, mechanical injury, mutation, toxins etc), but rather the process of evolution. Natural selection favors individuals that leave the largest number of surviving offspring, not necessarily those that live the longest. With advancing age after the onset of reproduction, natural selection weakens with the result that new alleles that impair health in later life are more likely to escape selection and accumulate in populations, causing aging (Medawar, 1952). The causes of this selection shadow can include extrinsic factors (e.g. predation, starvation) that increase mortality disproportionately in later life (Abrams, 1993; Hamilton, 1966; Moorad et al., 2019).

### 2.1 Antagonistic pleiotropy as a cause of aging

How might gene variants with bad effects on late-life health act? Here there are two ideas. First, they could be harmful mutations with effects that do not appear until late life. A suggested example is Huntington’s disease, which does not develop until mid-life, as the selection shadow deepens. This could explain its relatively high prevalence, despite being caused by a dominant mutation (Haldane, 1941). This instantiates the mutation accumulation theory of the evolution of aging (Medawar, 1952). Here, the harmful alleles that natural selection fails to eliminate and which cause aging are very much akin to disease-causing mutations, such as those causing Marfan syndrome or hemochromatosis. According to the mutation accumulation theory aging is a form of genetic disease, arising from a multiplicity of late-acting mutations.

**Fig. 1:**
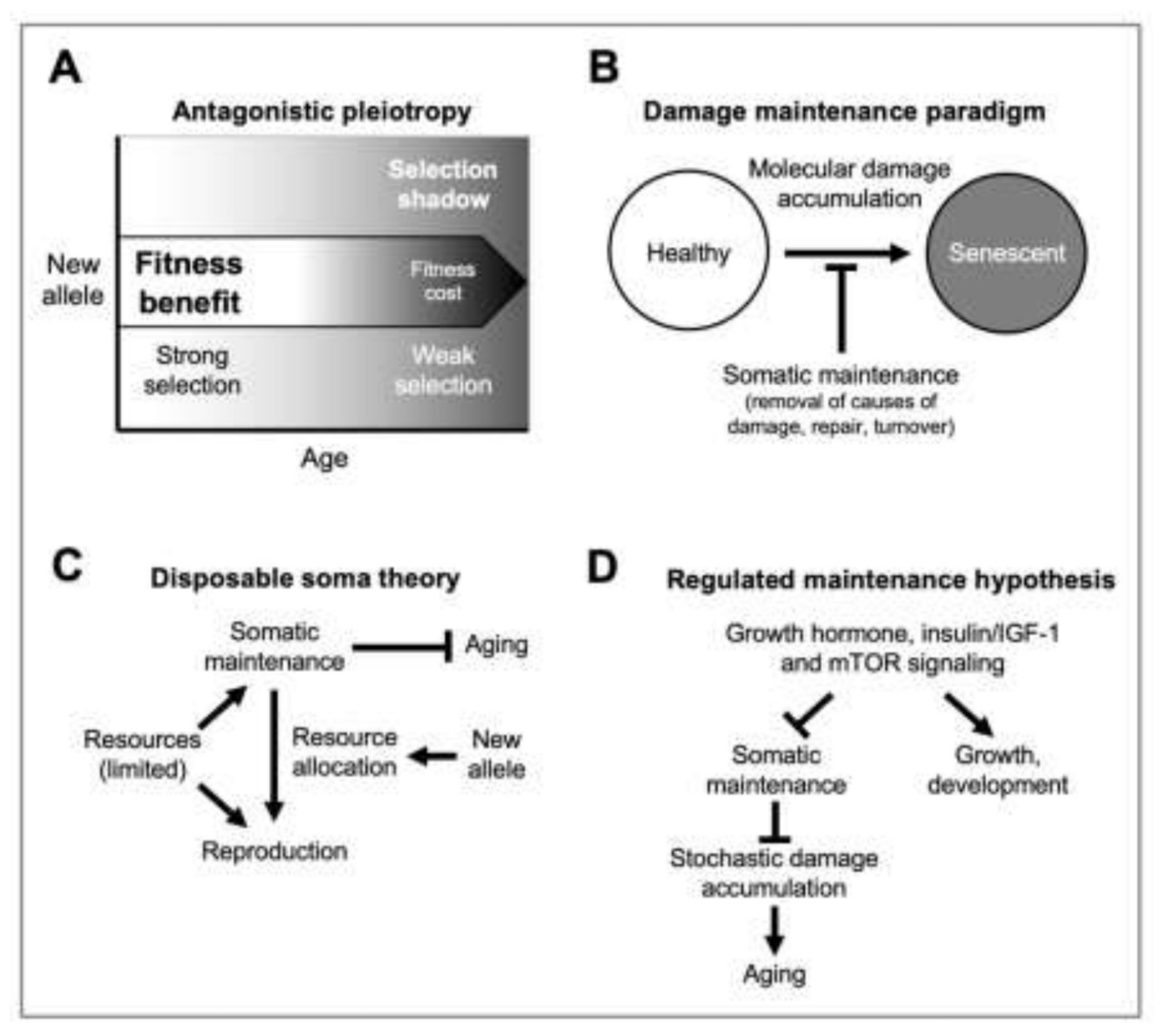
Ultimate and proximate mechanisms of aging (traditional interpretations). **A**, Antagonistic pleiotropy. A new allele that causes a fitness benefit in early life but a fitness cost (e.g. increased pathology) in later life may cause a net benefit in overall fitness due to the selection shadow (Williams, 1957). **B**, The damage/maintenance paradigm. Aging is caused by accumulation of stochastic molecular damage, whose levels can be controlled by somatic maintenance functions. **C**, The disposable soma theory. Investment of resources in reproduction more than somatic maintenance can increase fitness due to the selection shadow (Kirkwood, 1977). Genes promoting such resource allocation will exhibit antagonistic pleiotropy (Partridge and Gems, 2002a). **D**, Regulation of aging by nutrient pathways. Traditional view based on damage/maintenance paradigm (Partridge and Gems, 2006). The hyperfunction model argues that it is in fact the growth, development function that plays the main role in promoting aging.

Second, they may be gene variants that provide a fitness benefit in early life, but also promote pathology in later life. Due to the selection shadow, fitness benefits may outweigh the later detriment, resulting in the allele spreading through the population (Williams, 1957)(Fig. 1A). Here the new allele displays pleiotropy, i.e. has several effects, that are opposite in terms of impact on fitness, i.e. antagonistic pleiotropy (AP). Given that AP genes provide fitness benefits and are present due to positive selection, they cannot be viewed as defective or mutant; rather they are, in genetic terms, wild type. Thus, insofar as they are caused by AP genes, diseases of aging are genetic diseases arising from effects of the wild-type genome. The idea that normal gene function can cause disease might, from a medical perspective, seem a contradiction in terms. But evolutionary theory, now well supported empirically (Austad and Hoffman, 2018; Byars and Voskarides, 2020; Carter and Nguyen, 2011; Zhao and Promislow, 2019), tells us that normal genes can cause late-life pathology and diseases of aging. Such genes may be referred to as gerontogenes (Johnson and Lithgow, 1992).

This is a fundamental principle of pathophysiology critical for understanding late-life disease. Yet its utility is limited by the current lack of understanding of the actual biological mechanisms (biochemical, cellular, physiological) by which AP genes exert their effects. What is needed here is an account of the determinants of aging that encompasses both ultimate, evolutionary mechanisms and proximate, biological mechanisms into a single integrated understanding. Two such ultimate-proximate accounts of the causes of aging are the disposable soma theory and the programmatic theory.

## 3 Ultimate-proximate theory 1. Disposable soma: where damage causes aging

For many years a popular hypothesis about aging has been that its principal cause is the accumulation of random molecular damage (Harman, 1956; Shore and Ruvkun, 2013; Zimniak, 2008). The causes of molecular damage to biomolecules are diverse, ranging from DNA replication errors to glycation of proteins (Holliday, 1997; Rattan, 2008), but particular attention was paid to damage arising from reactive oxygen species (ROS) such as the superoxide (O_2_^-^) free radical produced as a by-product of oxidative metabolism (Beckman and Ames, 1998). If molecular damage causes aging it follows that the level of cellular maintenance that prevents damage accumulation is an important determinant of aging rate, and that the genetic determination of lifespan is likely to involve regulation of cellular maintenance mechanisms (Holliday, 1997; Partridge and Gems, 2006). In short, aging rate reflects the balance between stochastic damage accumulation and somatic maintenance levels (the damage/maintenance paradigm) (Fig. 1B).

The observation that cellular maintenance processes are costly in resource terms led to the deduction that trade-offs might exist between cellular maintenance and reproductive effort. Given limited resource availability in the wild, optimization of the trade-off would inevitably lead to levels of cellular maintenance below those necessary to entirely prevent damage accumulation. The result of this is a rapidly aging, disposable soma, in contrast to the immortal, non-aging germline (Kirkwood, 2005; Kirkwood, 1977) (Fig. 1C).

The disposable soma (DS) theory provides a clear and logical account of how trade-offs could arise between fitness traits at different points in the life history: early benefit (increased reproduction) and later cost (increased mortality due to gradual damage accumulation). Though not originally conceived as such (Armstrong, 2019) it provides a cogent explanation for the proximate mechanisms of AP gene action (Kirkwood and Rose, 1991). Yet despite its elegance, the DS theory remains unproven, and some experimental findings argue against it (Grandison et al., 2009; Piper et al., 2017; Zajitschek et al., 2019). One limitation is its dependence upon the damage/maintenance paradigm. While molecular damage, DNA damage in particular (Schumacher et al., 2021), is certainly a primary causal mechanism in some forms of senescent pathology (e.g. cancer), for many others this is far from clear (see below). While it is possible that DS effects could exacerbate pathologies where damage is a major driver, their importance as a major cause of aging currently seems unlikely (discussed further below) (Blagosklonny, 2007b; Blagosklonny, 2010e). But while its explanatory utility remains uncertain, as the first ultimate-proximate model, the DS theory was important in providing a template for the development of ultimate-proximate theories.

## 4 Ultimate-proximate theory 2. Where aging is programmatic

In the early 2000s it became increasingly clear that the insulin/IGF-1 signaling (IIS)/ mammalian (or mechanistic) target of rapamycin (mTOR) network can control aging rate across a range of organisms (Gems and Partridge, 2001; Kenyon, 2010). Discovering the IIS/mTOR-regulated, downstream mechanisms of aging itself promised to provide fundamental insights into the nature of senescence. Guided by contemporary beliefs, early attempts to understand this guessed that wild-type IIS accelerated aging by reducing somatic maintenance (e.g. antioxidant defense) thereby increasing reproductive output, consistent with the DS theory (Fontana et al., 2010; Gems and McElwee, 2005; Holzenberger et al., 2003; Honda and Honda, 1999; Kirkwood, 2005; Lithgow and Walker, 2002; Partridge and Gems, 2002b; Vanfleteren, 1993) (Fig. 1D). However, experimental findings during the same period were starting to raise doubts about the assumption that molecular damage is a primary cause of aging, particularly oxidative damage (Bjelakovic et al., 2007; Chong et al., 2007; Howes, 2006; Keaney and Gems, 2003; Keaney et al., 2004).

Against this background, two biogerontologists independently saw a way to rearrange existing concepts and findings into a new ultimate-proximate theory that is very different to DS. Although initial accounts of the theory, put forward by João Pedro de Magalhães (de Magalhães and Church, 2005) and Mikhail (Misha) Blagosklonny (Blagosklonny, 2006a), differ in emphasis and in detail, they have the same conceptual core (the programmatic theory). de Magalhães and Blagosklonny each put forward many ideas, some more persuasive than others; the following account of the framework of ideas centered on the programmatic theory is to a certain extent my own synthesis of their ideas.

### 4.1 The core programmatic theory

Growth hormone (GH), IIS and mTOR are part of a nutrient-sensitive signaling network that promotes growth, development and aging. This suggests that growth and development somehow cause aging. But three lines of reasoning initially seemed to argue against this deduction. First, it suggests that there is a program for aging, implying that aging is a purposeful adaptation; but according to evolutionary theory aging is non-adaptive, and therefore not programmed (Austad, 2004; Kirkwood, 2005; Partridge and Gems, 2002b). Second, growth and development are not obviously linked to somatic maintenance and molecular damage accumulation, which was assumed to be the main cause of senescence. Third, it is difficult to see how growth processes can limit lifespan. The programmatic theory provides answers to each of these three objections.

**Fig. 2:**
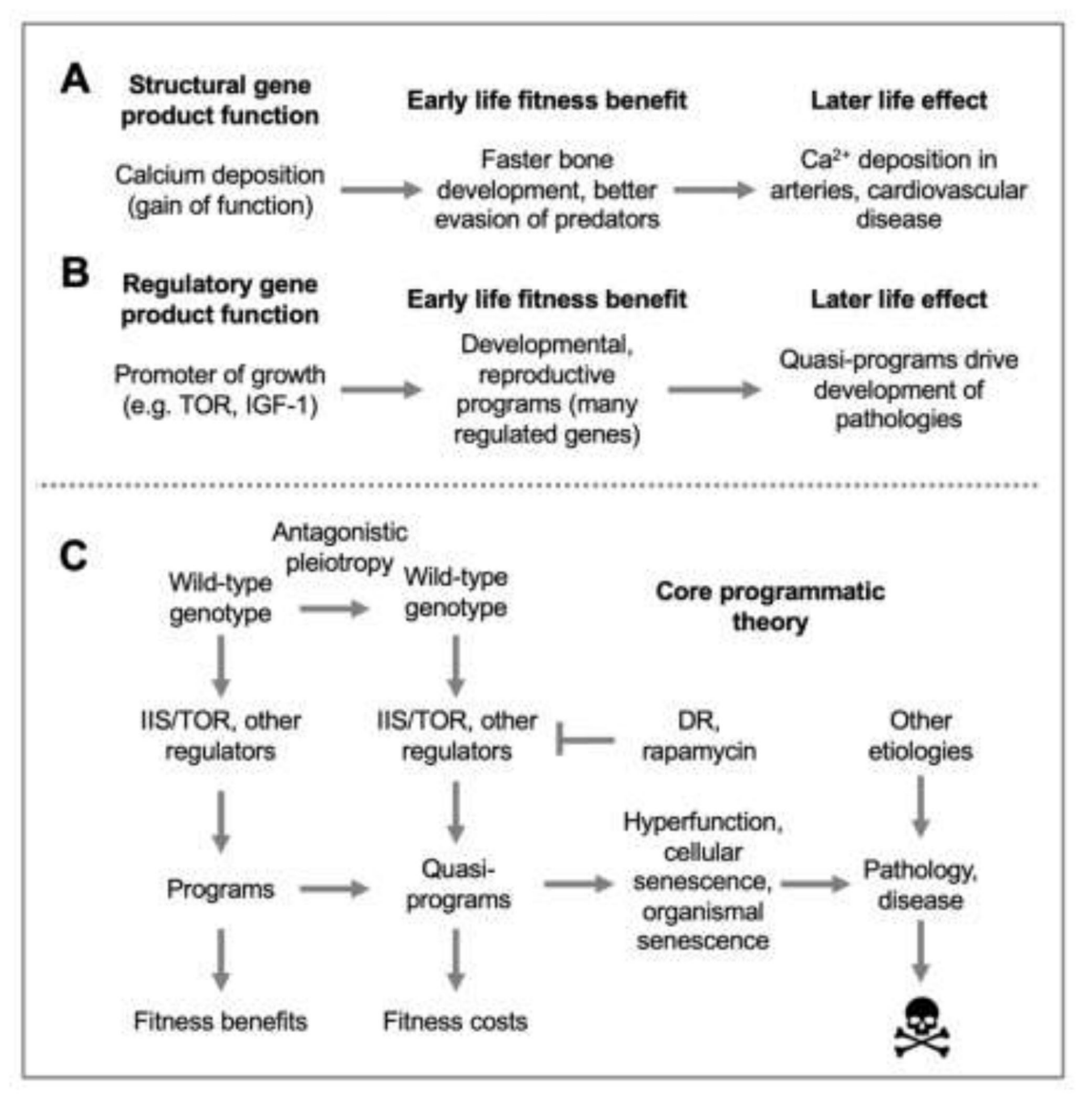
Antagonistic pleiotropy (AP) as hyperfunction. **A**, AP as run-on of structural gene function. Hypothetical example (Williams, 1957). **B**, AP as run-on of regulatory gene function. This results in pathogenic quasi-programs (Blagosklonny, 2006a). **C**, Overview of the core programmatic theory of aging (Blagosklonny, 2006a; de Magalhães and Church, 2005); here passage from left to right denotes advancing age. See glossary for definition of terms.

First, the program problem. Here it is helpful to consider Williams’ own initial thoughts about how AP might work at the level of gene function, which involve a hypothetical gene for calcium deposition. A new allele appears that increases Ca^2+^ deposition during bone development, and thereby promotes fitness (e.g. by aiding escape from predators); in later life, its continued action promotes Ca^2+^ deposition into blood vessel walls, contributing to arteriosclerosis (Fig. 2A) (Williams, 1957). In principle, a mechanism could evolve to switch off calcium deposition, but due to the selection shadow it does not. Here, an evolved function of a gene continues or runs on in a futile fashion in later life causing pathology. The principle involved here is notably different to the DS theory. If this can occur for the function of structural genes of this sort, why not for regulatory genes controlling entire developmental programs and involving action of large numbers of regulated genes (Fig. 2B)?

Evolutionary theory rules out biological programs *for* aging (i.e. aging as an adaptation) for most species, but not *programmatic* or *program-like* mechanisms that promote senescence (Blagosklonny, 2007c; de Magalhães and Church, 2005). Part of the confusion arises because the word *program* here has two meanings. On the one hand it can mean a genetically-determined and complex process involving changes in gene expression, cellular function, tissue composition etc (programmed in the *mechanistic* sense); on the other hand it can mean a program *for* something that promotes fitness (programmed in the *adaptive* sense) (Galimov et al., 2019). Aided by this disambiguation, the new theory is able to argue that in later life programmatic changes occur that are programmed in the mechanistic but not the adaptive sense, or as Blagosklonny describes them, *quasi-programmed* (Blagosklonny, 2006a). The derived noun *quasi-program*, describing a programmatic, pathogenic entity, is particularly helpful for discussion of programmatic pathophysiology.

To explain the quasi-program concept, Blagosklonny employs various homely analogies. For example, one wants hot water for tea and so boils some in a saucepan. If, having taken the water, one leaves the saucepan on the hot stove, the pan will become damaged. Here a purposeful program for preparing hot water becomes a futile quasi-program for damaging the saucepan (Blagosklonny, 2006a). In living organisms later-life off switches for developmental programs are often absent because of declining selection in later life (Blagosklonny, 2006a; Williams, 1957). Similarly, de Magalhães uses the analogy of a program to build a house, and imagines a carpet layer working on pointlessly after completion of the program: “ever-increasing layers of carpets will eventually prevent doors from opening, and ultimately, nobody will be able to get in or out of the house” (de Magalhães, 2012). This starts to explain how growth promotion by IIS/mTOR can promote aging: they promote both developmental programs and senescence-promoting quasi-programs (Blagosklonny, 2006a; de Magalhães and Church, 2005).

But what about molecular damage? de Magalhães and Blagosklonny both suggest that the assumption that aging is largely and primarily caused by accumulation of molecular damage is incorrect (Blagosklonny, 2006a; de Magalhães and Church, 2005). As Blagosklonny articulates it, the primary mechanisms of aging do not involve *loss* of function, but rather the opposite: too much function or, as he puts it, *hyperfunction*, driven by wild-type gene action. This claim challenges the entrenched assumption that aging is fundamentally a passive process of system failure and breakdown. Thinking of mTOR effects in particular, Blagosklonny argues that it is the opposite: an active, self-destructive process.

Finally, how can processes of growth and development lead to disease? Broadly speaking, the programmatic theory argues that developmental changes in adulthood, including late-life continuation of developmental programs, is pathogenic, causing disruption of tissue and organ function. Several examples of simple forms of developmental run-on follow. Presbyopia is a type of long-sightedness that increases with age. Its cause is continued, futile growth of the lens during adulthood, leading to a gradual increase in lens thickness (Strenk et al., 2005). In men, the prostate gland typically exhibits a gradual increase in size as a response to long term exposure to dihydrotestosterone. This leads eventually to benign prostatic hyperplasia (Nacusi and Tindall, 2011; Waters et al., 2000) and increased risk of prostate cancer, a major cause of age-related death in men (Untergasser et al., 2005). In a third example, this time hypothetical, the process of synaptic pruning in the brain that promotes cognitive development runs on in later life, leading to age-related cognitive decline (De Magalhaes and Sandberg, 2005). A final example affects babirusas, a type of wild pig found in Indonesia. Adult males have curved, tusk-like maxillary canine teeth that point backwards up over the snout. These continue to grow until in some cases the backward curve of their growth trajectory drives them into the cranium, sometimes piercing it through (Macdonald, 2018). Here, in each case, continuation of normal, wild-type growth leads to pathology. However, most aging-related diseases are etiologically multi-factorial, and contributions of programmatic etiologies more complex (described below).

A simplified scheme of the programmatic theory is shown in Fig. 2C. Beyond these core tenets, Blagosklonny and de Magalhães have each contributed additional ideas, which extend and strengthen the theorem. For example, Blagosklonny elaborates upon how quasi-programs affect lifespan and disease, and makes pointed critiques of various concepts arising from the damage/maintenance paradigm, while de Magalhães integrates the programmatic theory conceptually with earlier developmental theories of aging, and explores evolutionary mechanisms beyond AP and IIS/mTOR that contribute to programmatic aging. Ideas from these two commentators will be discussed in turn.

## 5 Mikhail Blagosklonny

### 5.1 Blagosklonny’s research approach

To understand Blagosklonny’s thinking it is helpful to know something about his background and approach. Unlike most biogerontologists, he has a clinical background (M.D. in internal medicine, Ph.D. in experimental medicine and cardiology). He is a proponent and practitioner of *conceptual research*, which uses explorations of the scientific literature to develop new hypotheses and to test them, sometimes repurposing existing data to draw new conclusions in ways unintended by the original researchers (Blagosklonny, 2007d; Blagosklonny and Pardee, 2002). He initially applied this approach for research in experimental oncology, including a focus on anti-cancer drug action (Blagosklonny, 2005a; Blagosklonny, 2005b; Blagosklonny, 2005c). An interest in the potential of the drug rapamycin as an anti-cancer agent (Blagosklonny and Darzynkiewicz, 2002) led him to the idea that this drug might act by inhibiting aging. This led in turn to the question of the causes of aging itself, and a re-examination of central ideas in biogerontology, both ultimate (e.g. antagonistic pleiotropy, disposable soma) and proximate (e.g. molecular damage accumulation, telomere shortening).

A strength of conceptual research is the relative ease of combining findings and concepts from different scientific disciplines, for example between basic and clinical research fields (Blagosklonny, 2003b), enabling broader insights. Blagosklonny’s conclusions draw extensively from both biogerontological sources and, importantly, clinical literature on age-related disease; thus, his ideas are more strongly grounded in contemporary research findings (including clinical findings) than in traditional theories about aging. They are also influenced by earlier ideas developed in Russia in the 1970-80s (Dilman, 1994) (discussed further below).

Given that the starting point of the work was to understand the action of rapamycin, his perspective is highly focused on this drug and the protein that it inhibits, mTOR (Blagosklonny, 2007a; Blagosklonny, 2010b; Blagosklonny, 2012c; Blagosklonny, 2014b; Blagosklonny, 2019a), which restricts its scope somewhat. However, his arguments are often applicable (and applied) to the broader endocrine and signalling network that regulates development and growth, including GH and IIS. In many of his discussions, the aging process that he describes is the set of processes controlled by GH/IIS/mTOR which, as he sometimes emphasises, is not the entire aging process.

Key to the initial development of Blagosklonny’s theorem was his realization that growth stimulation leads rapidly to cellular senescence when the cell cycle is arrested, with no involvement of molecular damage (Blagosklonny, 2003a). (Note here that cellular senescence, as defined by Hayflick, should not be confused with the broader phenomenon of senescence). His main ideas are set out in three early essays (Blagosklonny, 2006a; Blagosklonny, 2007b; Blagosklonny, 2008b), particularly the first of these. He then produced a very long series of follow-up essays (over 60), some derived from additional conceptual research with newer findings, and some responding to individual new papers. Many of these essays argue that an mTOR hyperfunction model better explains published findings than the conventional damage-based view, and often include explanatory reiterations of his version of the programmatic theory. The repetition to an extent reflects the lack of response to his work from other biogerontologists; “Repetitio est mater studiorum” as he notes at one point (Blagosklonny, 2018b). Some present new elaborations and additions to the theory, e.g. (Blagosklonny, 2007c; Blagosklonny, 2009b; Blagosklonny, 2010c; Blagosklonny, 2010d; Blagosklonny, 2018a; Blagosklonny, 2019b). For brief summaries of the content of 68 of his essays, see Table 1. The essays draw to some extent on his own laboratory work, mostly on the regulation of cellular senescence, see e.g. (Demidenko et al., 2009a; Demidenko and Blagosklonny, 2008; Demidenko and Blagosklonny, 2009; Demidenko et al., 2010; Demidenko et al., 2009b; Leontieva and Blagosklonny, 2016; Leontieva et al., 2015; Leontieva et al., 2012a; Leontieva et al., 2012b). At times they seem written in haste, as if painted with rough brushstrokes, but what they sometimes lack in polish they often make up for in originality and creative insight. Key elements of Blagosklonny’s framework of ideas, beyond the core programmatic theory include the following.

### 5.2 How do quasi-programs promoted by IIS/mTOR limit lifespan?

Aging is a deteriorative process that causes decline and death, but how? The core programmatic AP account provides a cause, but does not explain how it leads to life-limiting harm. It is here that Blagosklonny in particular sheds light, helped by three basic ideas that combine to give a very different view of aging to that which is traditional in biogerontology. First, that death due to aging is caused by disease. Second, that aging results from a nested series of life-limiting etiologies. Third, that programmatic AP promoted by IIS/mTOR causes life-limiting disease in a largely non-cell autonomous manner.

#### 5.2.1 Aging is pathology

**Fig. 3:**
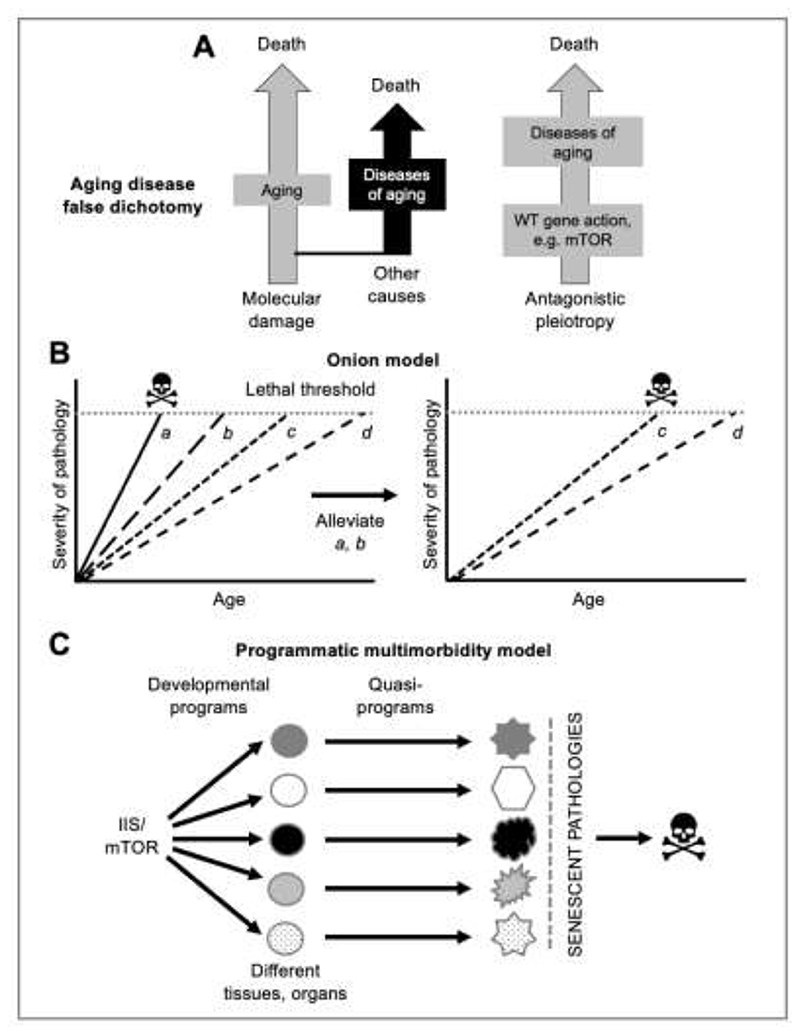
Concepts from the broader programmatic theory. **A**, The false distinction between aging and disease; adapted from (Blagosklonny, 2007b). Left, aging as a process distinct from late-life disease; here disease is incidental to understanding aging. Right, aging as diseases/pathologies caused by wild-type gene action; here disease/pathology is critical to understanding aging. Accounts of aging in terms of damage/maintenance tend to neglect the importance of disease in the aging process (Blagosklonny, 2012a; Gems and de Magalhães, 2021). **B**, The onion model of the relationship between aging, disease and lifespan (Blagosklonny, 2006a; de Magalhães, 2012). Lifespan is a function of one or more specific life-limiting pathologies, rather than any underlying process of aging as a whole. Left, lifespan is limited by pathology *a*. Right, a life-extending treatment acts by eliminating *a* and *b*. Lifespan is now limited by pathology *c*. **C**, How single genes can exert large effects on aging. Growth control pathways act in diverse tissues and organs, driving diverse programs and quasi-programs, the latter leading to diverse pathologies.

Biogerontologists usually use lifespan or mortality rate as metrics of aging rather than senescent pathology, and tend to view aging as a process distinct from age-related disease (Gems, 2015; Hayflick, 2007). By contrast, influenced by his medical training, Blagosklonny sees aging very much in terms of diseases: “No one dies from healthy senescence: humans and other mammals die from senescence-associated diseases such as cancer, stroke, myocardial infarction, [etc]” (Blagosklonny, 2006a). He argues that biogerontologists often have an erroneous view of lifespan as a function of damage, and diseases of aging as relatively incidental (Fig. 3A) (Blagosklonny, 2007b; Blagosklonny, 2012a). Surveying clinical literature that is typically beyond the biogerontological purview, he notes that many senescent pathologies and conditions are linked to mTOR over-activity, including cancer, atherosclerosis, hypertension, type 2 diabetes, osteoporosis, osteoarthritis, macular degeneration, and Alzheimer’s and Parkinson’s diseases (Blagosklonny, 2006a; Tsang et al., 2007). Thus, programmatic AP limits life by causing diseases of aging.

#### 5.2.2 Aging as a nested series of life-limiting etiologies

A tenet of the DS theory is that aging as a whole is caused by molecular damage accumulation, suggesting that enhanced somatic maintenance could prevent aging altogether (de Grey et al., 2002; Kirkwood and Rose, 1991). There do exist organisms for which this appears to be true: in the filamentous fungus *Podospora anserina*, reducing mitochondrial ROS production can prevent senescence entirely and produce a seemingly non-aging state (Dufour et al., 2000). By contrast, Blagosklonny views mTOR’s impact on lifespan not as an effect on aging as a whole but rather of promotion of multiple life-limiting pathologies (or multimorbidity). He reasons that if mTOR hyperfunction were suppressed (e.g. by inhibition with rapamycin) then other disease etiologies would become more life limiting (e.g. other quasi-programs, oxidative damage, mutation, telomere shortening) (Blagosklonny, 2006a; Blagosklonny, 2007b; Blagosklonny, 2021b)(Fig. 3B). Similarly, de Magalhães views programmatic etiologies as a “layer of mechanisms of aging” (de Magalhães, 2012). This suggests a view of aging as a nested series of causes of senescent pathology, rather like the layers of skin in an onion: the outer skin is the life-limiting etiology (IIS/mTOR), removal of which reveals a new layer (e.g. DNA damage, perhaps) underneath (Fig. 3B). Such an *onion model* is an implicit critique of the convention in biogerontology of viewing lifespan as a metric of aging overall; in fact, lifespan is a metric of life-limiting pathologies, which can vary with context (e.g. culture conditions, genotype, species)(Ezcurra et al., 2018; Gems and de Magalhães, 2021).

Inhibition of IIS/mTOR can increase lifespan substantially, suggesting an effect on aging as whole and, therefore, the existence of an overall aging process. An alternative view is that quasi-programs can arise independently in multiple tissues, promoting diverse senescent pathologies, all of which are supported by IIS/mTOR since they are fundamental drivers of growth and development (Fig. 3C). Thus, IIS/mTOR promotes senescent multimorbidity, but not the entire aging process. This suggests that the idea of “aging as a whole” was, in some sense, a conceptual Fata Morgana in the minds of biogerontologists produced by a combination of IIS/mTOR inhibition effects and theories such as DS.

#### 5.2.3 Non-cell autonomous action of quasi-programs

The idea that aging is caused by molecular damage suggests that senescence originates largely as a cell autonomous process, in which impairment of basic cell function leads to cell breakdown, and from there upward to tissue and organ failure. Hence, aging is a problem of cellular metabolism and cellular maintenance. By contrast, in the mTOR quasi-program model basic cell function is in many cases unimpaired; instead, cell function is changed in a way that disrupts tissue and organismal function. Pathology results from loss of tissue, organ and physiological homeostasis rather than of cellular homeostasis. Similarly, de Magalhães observes that one can broadly divide gene function into that specifying basic cell biology (respiration, DNA replication, repair) and that specifying developmental programs, and argues that the main causes of aging are located in the latter and not, as the damage/maintenance paradigm assumes, in the former (de Magalhães and Church, 2005).

Pathogenic effects of cellular hyperfunction are largely cell non-autonomous, involving hyperplasia, dysplasia, hypertrophy, hyper-secretion, atrophy and cell signal resistance (see below). Blagosklonny describes how many diseases of aging are caused by hyperfunction (Blagosklonny, 2006a; Blagosklonny, 2012a; Blagosklonny, 2013d). As one example, myocardial infarction results from a combination of factors, including coronary atherosclerosis and arterial spasm, hypertension, myocardial hypertrophy and thrombosis, for each of which hyperfunction is a contributory factor. Proliferation and hypertrophy of arterial smooth muscle cells (SMC) and lipid accumulation contributes to atherosclerosis; proliferation, hypertrophy, elevated contractile function and hyper-stimulation of arterial SMC all contribute to hypertension; mTOR hyperfunction contributes to cardiac hypertrophy; and increased aggregation and adhesion of platelets contributes to increased thrombosis. An example of hyperfunction causing atrophy is progression of osteoporosis due to osteoclast hyperactivity, which is promoted by mTOR (Blagosklonny, 2006a). Thus, the pathogenic effects of hyperfunction in the main do not work from bottom up, by harming individual cells that exhibit it; instead, hyperfunctional cells are robust and, in themselves, healthy, even though they might be damaged (as in cancer cells) (Blagosklonny, 2008b; Blagosklonny, 2012a). Instead, hyperfunctional cells cause pathology at the tissue and organ level. However, one noxious cell-autonomous effect of mTOR hyperfunction is reduced autophagic clearance of aggregation-prone proteins, increasing risk of several age-related neurodegenerative diseases (Blagosklonny, 2006a).

If pathogenic effects of cellular hyperfunction are largely cell non-autonomous, then what about aging in single cell organisms? Notably, in budding yeast (*Saccharomyces cerevisiae*) inhibition of TOR can increase lifespan (Blagosklonny and Hall, 2009), implying that TOR promotes cell death in this species. Blagosklonny suggests that aging in bacteria results from damage accumulation (Blagosklonny, 2012a), and presumably this applies to unicellular eukaryotes too.

#### 5.2.4 Programmatic etiologies as a target for intervention

This new model provides an account of rapamycin action against aging. Quasi-programs occur due to the late-life selection shadow, which has resulted in insufficient off switches (Williams, 1957); rapamycin in later life can rectify some of this insufficiency (Blagosklonny, 2006a). More broadly, the model predicts a general preventative approach to age-related disease: to allow enactment of programs but, in later life, inhibit quasi-programs. This possibility is implicit in Williams’ calcium gene scenario: one could imagine a treatment to prevent it from promoting vascular calcification in later life.

Validating anti-aging drugs requires biomarkers of aging, the search for which has been a long-standing objective in aging research. This includes a major program by the US National Institute on Aging initiated in 1988 to try to identify biomarkers of aging in rodents (Warner, 2004). Since aging is a developmentally-driven multimorbidity (rather than a metabolic problem within cells), then a good biomarker is progression of multiple diseases of aging (Blagosklonny, 2009b). Potential anti-aging drugs can be identified through re-analysis of outcomes of drug tests against individual diseases, to look for wider anti-disease effects. For example, rapamycin administered to renal transplant patients unexpectedly reduced cancer rates in several studies (Blagosklonny, 2009b). This principle implies that obesity accelerates aging, since it increases the rates of multiple aging-related diseases (Blagosklonny, 2011b), meaning that reducing obesity is an anti-aging treatment.

#### 5.2.5 Pathogenic effects of mTOR acting as a brake on growth

**Fig. 4:**
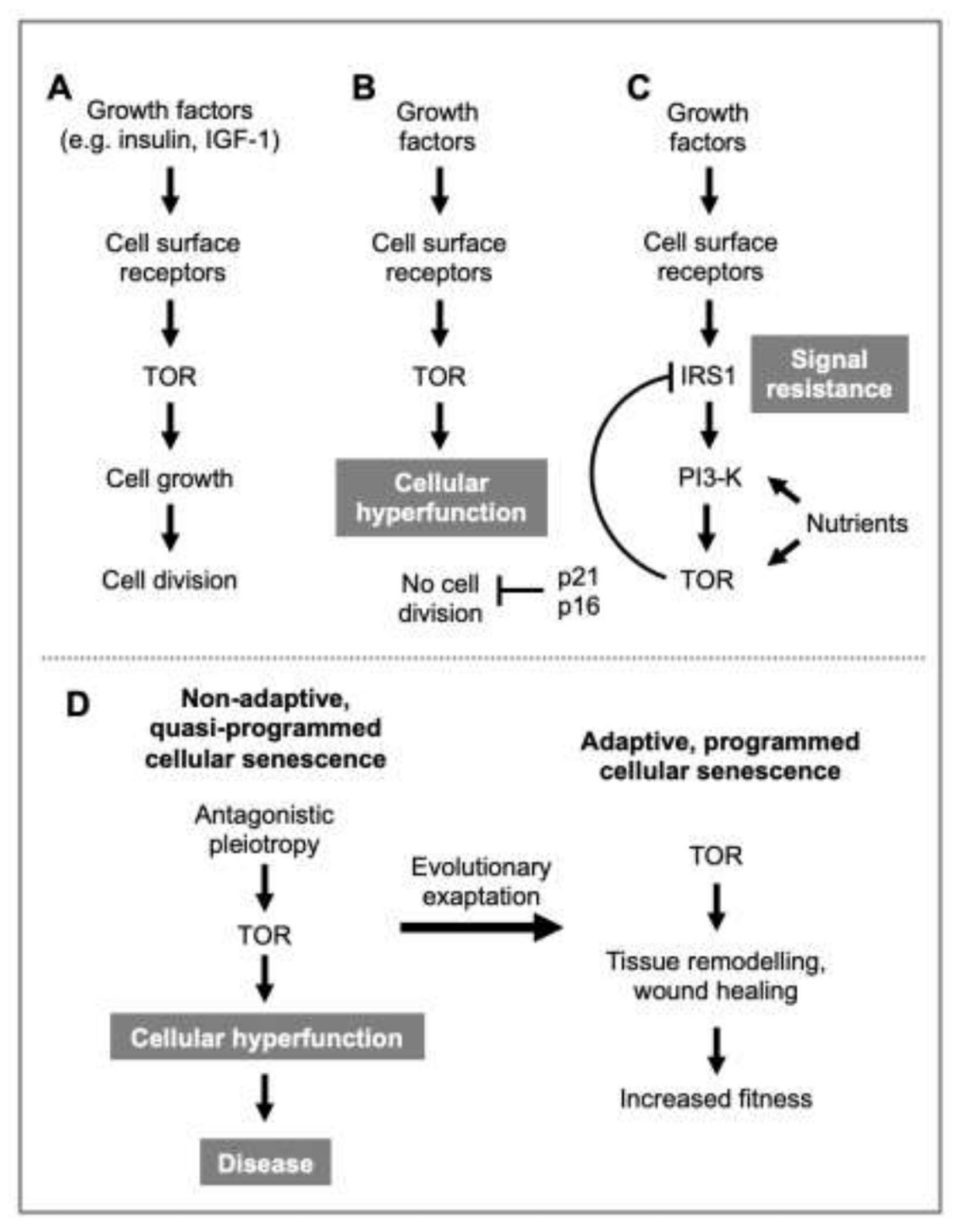
TOR in cellular senescence: evolutionary and mechanistic perspectives (Blagosklonny, 2006a). **A**, During development, cell growth and cell division occur in concert, leading to constant average cell size. **B**, When the cell cycle stops due to CDK inhibitor action, TOR stimulation continues and becomes hyperfunctional. **C**, Negative feedback inhibition of TOR occurs by phospho-inhibition of IRS1 (insulin receptor substrate 1) by the TOR effector S6 kinase; PI3-K, phosphoinositide 3-kinase. This reduces TOR hyperfunctionality by promoting signal resistance, which in turn can have pathogenic effects (e.g. insulin resistance, leading to type 2 diabetes). **D**, Evolution of adaptive, programmed cellular senescence by exaptation from non-adaptive quasi-programmed cellular senescence (hypothetical scheme). Most cellular senescence is pathogenic, caused ultimately by AP. But some forms of cellular senescence have evolved a function, as in wound healing promoted by senescent fibroblasts (Demaria et al., 2014). In the context of *adaptive* cellular senescence, the term “senescence” is illogical.

A recurrent theme in Blagosklonny’s thinking is that mTOR is a driver of growth that becomes pathogenic when growth has to cease (Fig. 4A,B). mTOR itself can act as a brake on growth by cutting off upstream growth-promoting signals via feedback inhibition. Specifically, mTOR acts through S6 kinase to inhibit IRS-1 (insulin receptor substrate) (Fig. 4C), leading to receptor signal resistance, including insulin resistance that contributes to type 2 diabetes (Blagosklonny, 2006a). Signal resistance caused by mTOR may also contribute to age-related loss of stem cell function (Blagosklonny, 2008a).

Blagosklonny views mTOR within the cell as a microcosm at the cellular level of homeostatic function at the organismal level, particularly in the hypothalamus, where pathology also develops due to signal resistance (Blagosklonny, 2013b; Dilman, 1971). As he points out, like the hypothalamus, mTOR integrates diverse signals, in the latter from insulin, mitogens, cytokines, oxygen, and nutrients. The recurrence of AP behavior of mTOR suggests the presence of a trade-off between the risks of allowing vs preventing growth that hinges on mTOR activity. The idea that a brake on growth at the end of ontogenesis causes aging was proposed previously, in a different context (Bidder, 1932).

Blagosklonny also emphasizes that cellular senescence occurs as a pathogenic consequence of mitogenic signals acting when cell cycle progression is blocked, leading to mTOR-driven geroconversion to the hypertrophic, hypersecretory state (Blagosklonny, 2003a; Blagosklonny, 2006a; Blagosklonny, 2006b; Blagosklonny, 2009a; Blagosklonny, 2011a; Blagosklonny, 2012b; Blagosklonny, 2014a; Blagosklonny, 2018b; Blagosklonny, 2021a). However, cellular senescence as a quasi-programmed, non-adaptive abnormality occurring during aging is, one assumes, distinct from programmed, adaptive cellular senescence with functional utility in embryogenesis or wound healing (Demaria et al., 2014; Munoz-Espin and Serrano, 2014). The importance of mTOR in both cases suggests that adaptive cellular senescence could have evolved by exaptation (acquisition of a new function by an existing feature) (Gould and Vrba, 1982) from a non-adaptive, purely pathological ancestor (Fig. 4D).

#### 5.2.6 Programmatic loosening as a feature of aging

According to programmatic theory, the processes of development and aging unfold as the result of shared developmental mechanisms. But compared to development proper, aging is far more variable and, seemingly, disordered. Development is a highly precise, stereotyped process. By contrast, each person’s individual experience of aging is unique; one starts with lower backpain and shingles, and progresses to macular degeneration and death from colon cancer, while another starts with osteoarthritis in the fingers and urge incontinence, and moves on to vascular dementia and an eventual lethal stroke. To some extent this variation will reflect differences in genotype and environment. The importance of factors other than genetic difference is illustrated by the considerable degree of discordance even between monozygotic twins in terms of diseases of aging, ~50% for type 2 diabetes, for example (Poulsen et al., 1999). Even in *C. elegans* where population cohorts of genetically identical individuals can be maintained in the same Petri dish, the lifespans of individual nematodes vary greatly (from ~10-30 days under standard culture conditions), and different causes of death can be observed (Rea et al., 2005; Zhao et al., 2017).

Variability in outcome would seem to be an inherent property of the aging process. Blagosklonny argues that variability of outcome is a characteristic feature of quasi-programs: “As an unintended continuation of the developmental program, the quasi-program is not very precise” (Blagosklonny, 2007b). Consistent with this, chronological age is a good surrogate for biological age (as estimated using a methylation clock, DNAm age, described below) during development but increasingly less so in later life (Horvath and Raj, 2018). Moreover, gene expression heterogeneity remains low during development but increases during aging (Isıldak et al., 2020). The authors of that study interpreted this as evidence that stochastic rather than developmental processes contribute to aging; an alternative interpretation is that such heterogeneity reflects developmental stochasticity characteristic of quasi-programs.

The progressive developmental loosening associated with quasi-programs may result from the age decline in natural selection. In earlier life greater selection maintains the precision of developmental programs, but it wanes with increasing age, this precision declines. A formal possibility is that genetic determinants of precision are subject to antagonistic pleiotropy involving trade-offs between earlier developmental precision and later precision loss.

In summary, the programmatic theory defines an etiological principle that contributes to many diseases of aging. Wild-type gene action promotes pathogenic quasi-programs in later life, in line with Williams’ original conception of AP. Promotion of quasi-programs by wild-type levels of IIS/mTOR can at least partially explain how they accelerate aging. This conceptual framework also provides a principle for treatment (inhibition of quasi-programs), actual possible treatments (e.g. mTOR inhibitors), and approaches to test them experimentally in humans.

### 5.3 Blagosklonny on the attack

In many of his essays, Blagosklonny uses conceptual research to compare how well damage-based and programmatic theories do at explaining findings past and present. Overall, these find in favor of the latter, and support critiques of several traditional biogerontological claims and concepts, which he sometimes pokes fun at for their perceived inadequacies.

#### 5.3.1 Against the oxidative damage theory

The theory that molecular damage caused by ROS causes aging dominated biogerontology from the 1980s to the mid-2000s. The wide net of Blagosklonny’s conceptual research led him to conclude that scientific evidence does not generally support the ROS theory (Blagosklonny, 2006a; Blagosklonny, 2008b), a suspicion shared by de Magalhães and I (de Magalhães, 2005; Keaney and Gems, 2003; Keaney et al., 2004). The crisis in the ROS theory that culminated in 2008-09 has been reviewed extensively elsewhere (Gems and Doonan, 2009; Perez et al., 2009; Shields et al., 2021; Van Raamsdonk and Hekimi, 2010). It is true that there is a correlation between aging and accumulation of molecular damage. Of this, Blagosklonny says: “With age, I have accumulated a considerable knowledge regarding the ROS theory. Does this knowledge cause my aging? I hope not” (Blagosklonny, 2008b). Instead, he argues that molecular damage accumulation is symptomatic of later stages of diseases of aging caused by hyperfunction.

#### 5.3.2 Against disposable soma

The mTOR-driven quasi-programs (mTOR/QP) model provides an alternative ultimate-proximate model to the disposable soma (DS) theory, that is closer to Williams’ own original idea about proximate mechanisms of AP. Blagosklonny even refers to this model as “disposable soma theory 2”, insofar as the soma is disposable as the result of mTOR/QP action (rather than resource allocation etc) due to AP and the later-life selection shadow (Blagosklonny, 2013c). He points out that the mTOR/QP model originates from consideration of diverse scientific findings, unlike either Harman’s free radical theory (Blagosklonny, 2008b) or the DS theory (Blagosklonny, 2010e), and argues that it outperforms DS at explaining a range of phenomena.

**Fig. 5:**
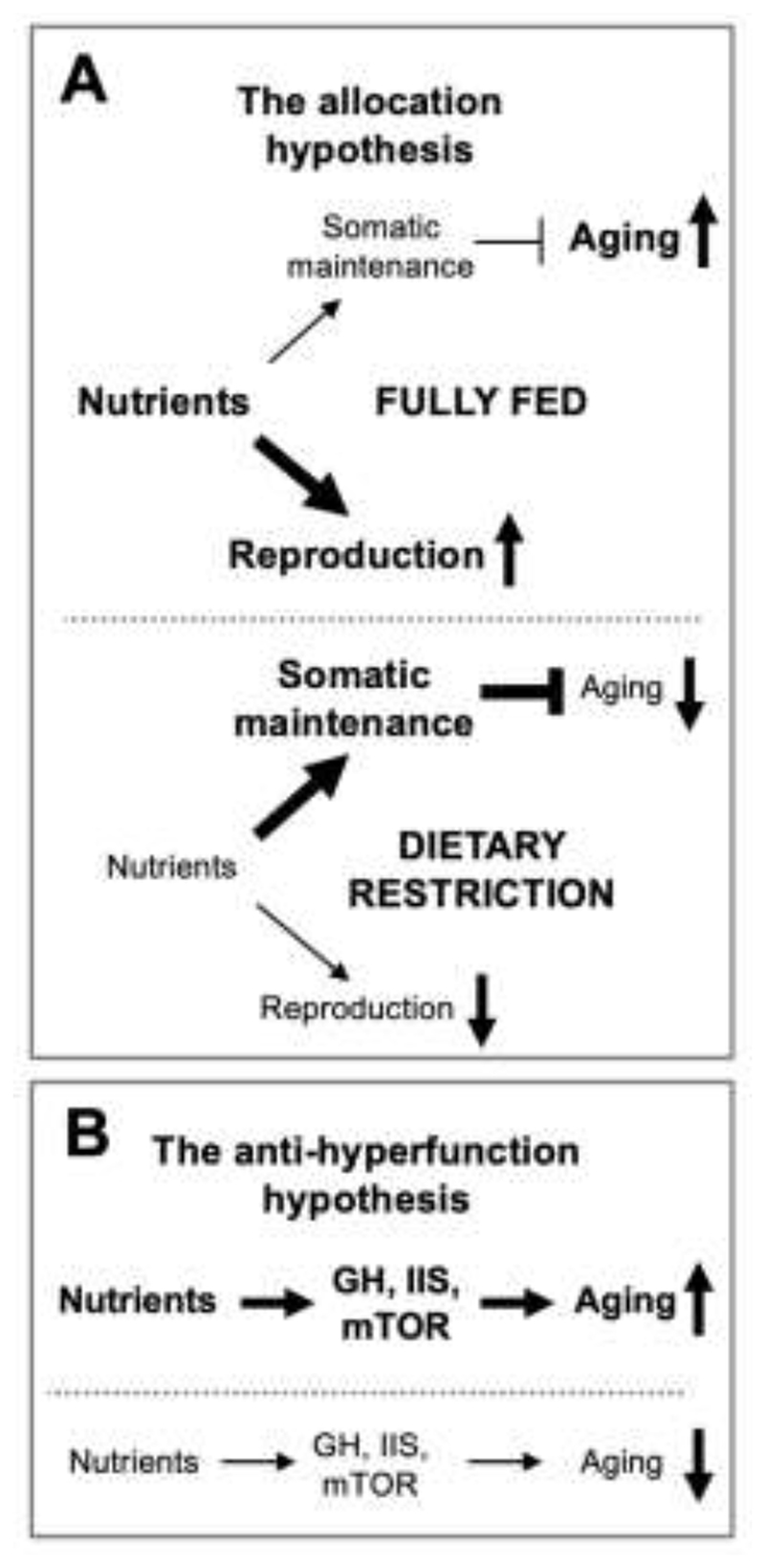
Alternative theories about how dietary restriction increases lifespan. **A**, The allocation hypothesis, based on the disposable soma theory. Reduced food leads to increased investment in somatic maintenance. **B**, Explanation based on the hyperfunction theory. Here reduced nutrients reduces growth pathway signaling, thus reducing quasi-programmed hyperfunction. Figure redrawn from (Blagosklonny, 2007b). The latter is both more parsimonious and consistent with existing knowledge of pathophysiology of various diseases of aging.

For example, the life-extending effects of dietary restriction (DR) have been reconciled with DS by the allocation hypothesis. Here it is argued that DR leads to diversion of resources from reproduction to somatic maintenance, thereby increasing lifespan (Holliday, 1989; Kirkwood and Shanley, 2005; Masoro and Austad, 1996)(Fig. 5A). Blagosklonny argues that this is implausible for a number of reasons. Perhaps most cogently, a more parsimonious prediction from DS is that increasing resources for somatic maintenance would increase lifespan and vice versa (Blagosklonny, 2007b). By contrast the mTOR/QP model argues simply that reduction in nutrients reduces mTOR activity, which suppresses both growth and QPs (Fig. 5B).

As another example, inhibition of protein biosynthetic genes in *C. elegans* increases lifespan, and it was suggested that this is due to freeing up of resources that are then invested in somatic maintenance (Hansen et al., 2007; Hipkiss, 2007). Blagosklonny argues instead that reducing protein synthesis prevents QP progression, pointing out that mTOR promotes protein synthesis (Blagosklonny, 2007b). As a third example, it was argued from the DS theory that women live longer due to increased investment into somatic maintenance (Kirkwood, 2010). Blagosklonny reasons that DS predicts that women should live shorter, since they invest more into reproduction; he suggests instead that increased mTOR activity promotes muscle growth in males, but accelerates aging (Blagosklonny, 2010a; Blagosklonny, 2010d; Leontieva et al., 2012b).

#### 5.3.3 Against stress-response hormesis

For some toxins, a lower dose range exists that promotes health (e.g. increased growth rate, stress resistance), an effect known as hormesis (Calabrese et al., 1999). In some cases this occurs due to induced expression of cytoprotective genes, so called stress-response hormesis (Gems and Partridge, 2008). For example, sub-lethal heat stress induces a heat shock response that can protect *C. elegans* against subsequent heat stress that would otherwise be lethal (Lithgow et al., 1995). Treatments that chronically increase ROS levels can increase *C. elegans* lifespan (Schulz et al., 2007; Yang and Hekimi, 2010), seemingly a challenge to the ROS theory. However, it has been suggested that this could reflect hormesis, where induction of antioxidant defences by ROS protects against aging caused by ROS; this includes ROS production by mitochondria inducing mitohormesis (Radak et al., 2005; Ristow and Zarse, 2010). Blagosklonny sees this account as an attempt to rescue the ROS theory from findings that contradict it, and sends up its logical awkwardness (increasing ROS reduces ROS). He compares it to Baron Munchausen’s adventure when, falling into a swamp, he escapes by pulling himself up by his own hair (Blagosklonny, 2008b). He suggests instead that effects of stress on lifespan more plausibly result from inhibition of growth processes that drive both development and quasi-programs (Blagosklonny, 2007b).

A constraint to progress in biogerontology is the ever-increasing volume of research output, which means it is difficult for researchers to keep abreast of the whole field. For this reason, the conceptual research approach is timely. By using it Blagosklonny has been able to develop a coherent and empirically well-supported framework of understanding of many aspects of the biology of aging. Yet it by no means presents a full picture of the process of aging, nor does it pretend to.

## 6 João Pedro de Magalhães

The programmatic theory reconciles a mechanistic theory based on developmental change with the AP theory, and provides an account of IIS/mTOR action on aging. But both developmental changes during aging, and AP are wider phenomena, as has been emphasized (Maklakov and Chapman, 2019), and all causes of aging is a wider category still. de Magalhães, originally from Portugal and now at the University of Liverpool, gives some consideration to programmatic etiologies in the broader sense (de Magalhães and Church, 2005), and explores issues relating to the programmatic theory that Blagosklonny touches on more briefly or not at all, including the following. How does the programmatic theory relate to earlier developmental theories of aging? And: how does it relate to epigenetic changes during adulthood?

### 6.1 From developmental theories to the programmatic theory

Over the years it has often been suggested that aging is a form of developmental abnormality, resulting e.g. from increased or decreased gene silencing, transcriptional deregulation, dysdifferentiation or overdifferentiation (de Magalhães and Church, 2005). Certain broad characteristics of aging are suggestive of its developmental nature. For example, different mammals (e.g. mice vs humans) differ greatly in terms of rate of development, timing of maturity, and of appearance of many of the same diseases of aging (e.g. cancer, osteoporosis, cataracts) (de Magalhães and Church, 2005). This is also true of the gradual age-dependent decline in cognitive function (De Magalhaes and Sandberg, 2005). Moreover, across mammalian species the ratio between time from conception to sexual maturity and adult lifespan is approximately constant, at about 1:4 (Charnov, 1993), suggesting a possible mechanistic relationship between development rate and adult lifespan (de Magalhães and Church, 2005). Similarly, the complex and stereotyped nature of many manifestations of senescence (e.g. atherosclerosis, osteoporosis, male pattern baldness) seem more consistent with programmatic pathophysiology than random molecular damage (Blagosklonny, 2006a).

While it is clear that both stochastic damage and programmatic changes contribute to senescence, in terms of their relative importance these are competing theories. The former predominated during recent decades, but less so prior to that. For example, as de Magalhães points out (de Magalhães, 2012), the discovery of effects of DR on aging, initially in fruit flies and rats, arose from the idea that slow growth rate results in slower aging (McCay and Crowell, 1934; Northrop, 1917; Osborne et al., 1917; Park, 2010), including Bidder’s hypothesis (Bidder, 1932). Later work showing that although life-long DR limits growth, adult-limited DR was sufficient to extend lifespan in fully grown mice (Weindruch and Walford, 1982) appeared to argue against the developmental theory; however, it is consistent with stunting of quasi-programs during adulthood.

Similarly, in *chico* (insulin receptor substrate) mutant fruit flies with reduced IIS, increased lifespan is seen in both *chico* homozygotes and heterozygotes, but only the homozygotes exhibit reduced body size (Clancy et al., 2001; Tu et al., 2002). Thus, *chico* mutant longevity is not caused by their reduced body size (Gems and Partridge, 2001), suggesting that reduced growth does not slow aging; however, these results, again, do not rule out suppression of quasi-programs during adulthood. In fact, *chico^-/+^* heterozygote females show reduced egg production (Clancy et al., 2001), consistent with biosynthetic (and programmatic) insufficiency.

Arguably, the confusion here stems partly from ambiguities arising from the words *growth* and *development*. Simply understood these refer to changes occurring during ontogenesis that largely cease at sexual maturity. A metric of growth is adult body size, and to speak of *growth* after maturation in organisms with determinate growth, after they have stopped growing, makes little sense. But in the programmatic model, growth pathways promote quasi-program progression, or “twisted growth” as it has been described (Blagosklonny, 2008b) during adulthood. Similarly, *development*. A quasi-program is defined as “a program for development that has not been turned off” (Blagosklonny, 2006a). Yet it is clear that what is meant here is development in the sense of all complex programs involving growth and differentiative change: not only those of ontogenesis, but also for example those involved in reproduction and tissue repair and remodelling during adulthood (c.f. the role of “senescent” fibroblasts in wound healing) (Demaria et al., 2014). To avoid this confusion the term *programmatic theory* is used here, rather than *developmental theory* as previously (Maklakov and Chapman, 2019). This avoids confusion with earlier developmental theories of aging, and over the meaning of *development*, and also alludes to the reconciliation of ultimate and proximate perspectives in the theory.

### 6.2 From the programmatic theory to epigenetic aging and biological clocks

A recent topic of interest among biogerontologists has been epigenetic changes during adulthood (Benayoun et al., 2015; Horvath and Raj, 2018). de Magalhães notes studies showing similarities between changes in gene expression occurring during development and aging (Lui et al., 2010; Somel et al., 2010; Takasugi, 2011) as evidence that drivers of epigenetic change are programmatic rather than stochastic (de Magalhães, 2012). As a developmental process, quasi-program execution is expected to involve epigenetic change. Thus, the programmatic theory provides an explanation in terms of ultimate and proximate causes for epigenetic changes in aging (though this does not rule out the action of other contributory mechanisms).

The question of how biological time is marked during aging is central to biogerontology, and many forms of clock have been proposed. These include a metabolic clock in the rate-of-living theory (Pearl, 1928), a telomere shortening replicometer in replicative senescence (Olovnikov, 1996), and epigenetic changes such as DNA methylation (Horvath, 2013; Horvath and Raj, 2018). According to the programmatic theory, the major aging clock is a developmental one. de Magalhães sees epigenetic changes as likely to arise from the ticking of a developmental clock (de Magalhães, 2012), whose speed is increased by IIS/mTOR (de Magalhães and Church, 2005). Consistent with this, the methylation clock is slowed by conditions in which mTOR is inhibited (Blagosklonny, 2018a), for example in mice treated with rapamycin, in long-lived Ames (*Prop1*), Snell (*Pit1*), and Laron (*Ghr*) dwarf mutant mice with defective GH signaling (Cole et al., 2017; Consortium, 2021; Petkovich et al., 2017; Wang et al., 2017), and in rapamycin-treated cultured human fibroblasts and keratinocytes (Horvath et al., 2019; Matsuyama et al., 2019). Moreover, mTOR functions in circadian clocks, underscoring the role of mTOR in setting the pace of biological time (Blagosklonny, 2018a).

The developmental basis of epigenetic changes during adulthood in mammals has received further support from recent work by Steve Horvath and colleagues. Of particular note, methylation clocks mark time during embryogenesis and postnatal development as well as (running much more slowly) during adulthood, and clock methylation sites are strongly associated with genes specifying developmental processes. The latter include those under regulation of the polycomb repressor complex (PRC), including multiple Hox genes, leading to the conclusion that “the essence of the aging process itself is an integral part of, and the consequence of the development of life” (Raj and Horvath, 2020). The link between clock methylation sites and PRC-regulated genes was also recently seen in a universal mammalian methylation clock, implying evolutionary conservation of developmental clock mechanisms (Consortium, 2021).

Almost a century ago, Raymond Pearl attributed rate-of-living effects, as seen in effects of ambient temperature on lifespan in poikilotherms (e.g. *Drosophila*, *C. elegans*)(Klass, 1977; Loeb and Northrop, 1917) to metabolic rate (Pearl, 1928), an idea that was subsequently linked to the ROS theory (Sohal and Weindruch, 1996). The programmatic theory suggests instead that effects of temperature on both development and aging reflects a change in the rate of developmental processes.

## 7 Vladimir Dilman: programmatic aging within a multifactorial model

As emphasized in the opening of this essay, antagonistic pleiotropy and programmatic mechanisms are clearly not the sole cause of aging. To close, I will present a sketch of a model of the whole aging process, derived from a scheme originally developed in the 1980s by Vladimir Dilman, but modified here in the light of recent advances. Dilman, who died in 1994, was a preeminent gerontologist and clinician in the U.S.S.R., in many ways the Soviet Union’s equivalent to thoughtful biogerontologists in the West such as Alex Comfort, Bernard Strehler, Richard Cutler and Caleb Finch, but his theories are little known outside Russia. He was also the father of Misha Blagosklonny.

### 7.1 Dilman’s ontogenetic and four models theories

**Fig. 6:**
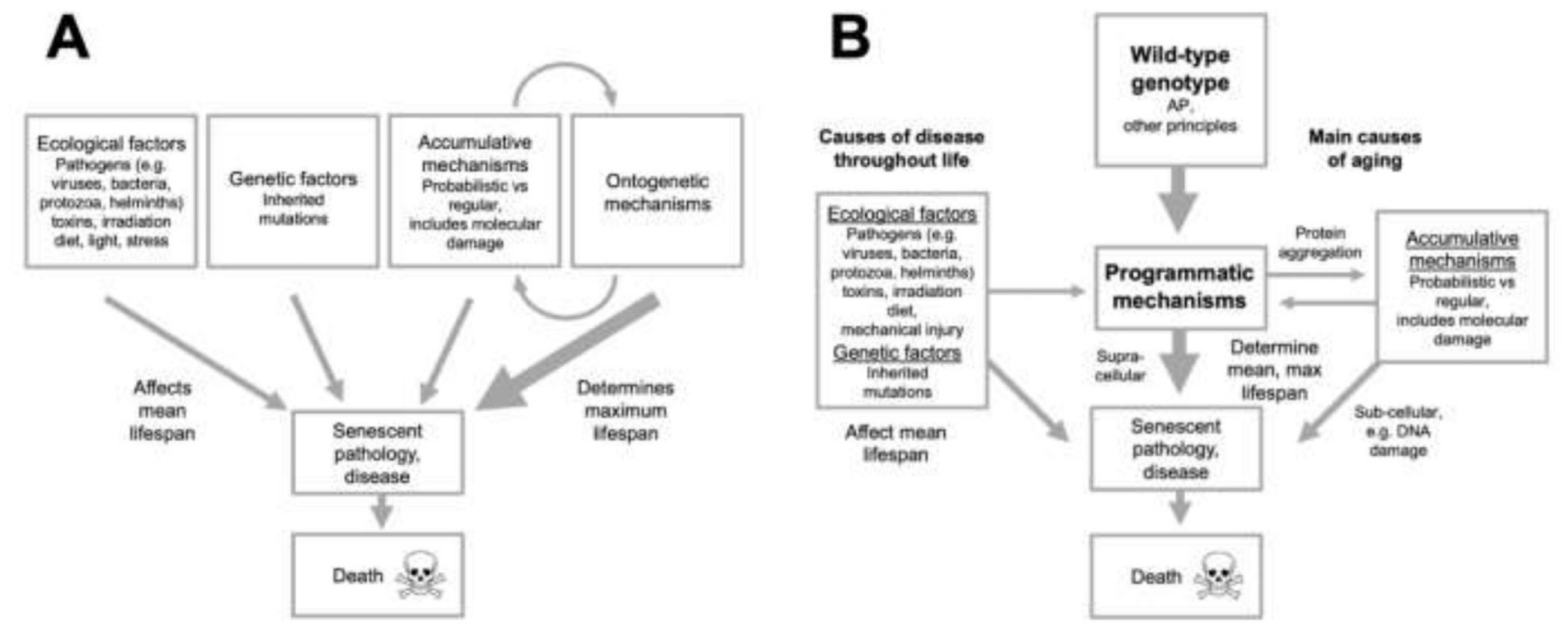
The bigger picture. Multifactorial models, in which programmatic AP is a single element. **A**, Dilman’s four models mechanism. This representation is based on his account, but leaves out his hypothalamic threshold model (Dilman, 1994). Here the major determinant of aging and mean and, particularly, maximum lifespan is ontogenetic mechanisms, with accumulative mechanisms (e.g. molecular damage accumulation) playing a lesser role. Ecological (i.e. extrinsic) and genetic determinants contribute to senescent pathology, and are a major determinant of mean lifespan, and variation in individual lifespan. **B**, Dilman’s multifactorial model rearranged, to incorporate more recent ideas. Here the major determinant of programmatic (ontogenetic) senescence is wild-type genotype, including pathogenic wild-type gene action due to antagonistic pleiotropy. But genotype-specified programmatic mechanisms contributing to aging clearly involve more than AP (Maklakov and Chapman, 2019). The model makes clear the centrality of wild-type function in aging. Even in the absence of other factors (ecological, genetic), senescence will occur, and maximum lifespan will change little. Factors that contribute to disease throughout life and do not reflect wild-type function, both extrinsic (ecological) and intrinsic (mutation) are grouped into one broad category. Included here is mechanical damage (mechanical senescence), not included by Dilman. One determinant not shown is the effect of extrinsic factors on damage accumulation (e.g. skin damage due to solar UV radiation).

Dilman argues that, overall, aging and age-related disease are etiologically multifactorial, and attributable to four distinct disease models: ecological, genetic, accumulational and ontogenetic (Dilman, 1994) (Chapter 11) (Fig. 6A). *Ecological* here is in the sense used by Frederic Ludwig to describe the main disease model in medicine, where diseases result from harmful extrinsic factors ranging from infectious pathogens to dietary toxins (Ludwig, 1980). *Genetic* here refers to inherited genetic diseases, rather than somatic mutations or pathogenic action of wild-type genes. The *accumulational* model includes molecular damage accumulation, but also other types of pathogenic accumulative processes. The *ontogenetic* model involves the continued action of developmental processes in later life promoting senescence.

According to this four models scheme, mean lifespan and inter-individual variation in lifespan is largely attributable to ecological and genetic determinants, and maximum lifespan to accumulational and, particularly, ontogenetic determinants. While Dilman’s ontogenetic model clearly prefigures the programmatic theory, it was derived as a generalization from a now partially outdated theory about the control of aging by the hypothalamus. Briefly, this argued that aging is largely caused by programmed loss of hypothalamic sensitivity to feedback inhibition, leading to compensatory endocrine changes with pathogenic consequences, what he called *hyperadaptosis* (Dilman, 1984). The ontogenetic processes that he particularly refers to are those controlling systemic homeostasis in adulthood, namely energy metabolism (insulin), reproduction (estrogen) and adaptive systems (glucocorticoids).

Yet in amongst Dilman’s many ideas and reflections are key elements of the new programmatic theory, including the following. The ontogenetic model itself is prescient; as Dilman describes it: “the genetic program of an organism is not constructed according to the rule ‘from beginning to end,’ but proceeds ‘from a beginning, with the end unspecified’” (Dilman, 1994) (Chapter 6). He notes in passing the congruence between the ontogenetic model and antagonistic pleiotropy (Dilman, 1986). Like Blagosklonny, he emphatically rejects the distinction drawn between normal aging and age-related disease: “normal aging is a disease, or more precisely, a sum of diseases” (Dilman, 1994) (Chapter 6). Like de Magalhães, he emphasizes that ontogenetic etiologies are operative at a level above that of the cellular; however, he views the key level as systemic, while in the modern programmatic theory it is at all supra-cellular levels (from tissue microenvironment to systemic). By contrast, at the subcellular level, accumulative mechanisms are more important (e.g. DNA damage accumulation).

Again like de Magalhães, he views the fixity among mammals of the length of the developmental period as a proportion of total lifespan as support for the role of ontogenetic mechanisms in aging. Here he makes an interesting observation. The standard explanation for this constancy is that an increased selection shadow (e.g. due to increased extrinsic mortality) leads to the evolution of earlier sexual maturity and shorter lifespan, via independent mechanisms (earlier reproduction increases fitness, earlier selection shadow allows earlier senescence to evolve) (Charnov, 1993; Harvey and Purvis, 1999; Promislow, 1993). But Dilman wonders about the obverse situation, where longevity evolves to increase fitness via a longer reproductive span. He asks: given the independent mechanisms model, why should a delay in sexual maturity occur? More plausibly, longevity evolves through deceleration of the entire ontogenetic program, which slows both development and aging (Dilman, 1994) (Chapter 11); Blagosklonny later made a similar point (Blagosklonny, 2013a). According to this interpretation, the longer development time in humans relative to other higher primates evolved not due to any fitness benefit, but as a side-effect of selection for increased longevity, i.e. it is an unselected outcome of antagonistic pleiotropy, an evolutionary spandrel (Gould, 1997; Gould and Lewontin, 1979). Consistent with the ontogenetic model is the earlier puberty, accelerated cognitive decline and shorter lifespan in mice over-expressing growth hormone, and the converse effects in dwarf and DR mice (Chandrashekar et al., 2004).

A major question raised by multifactorial models of aging is how the component factors interact with one another and here Dilman is, again, insightful. He argues that both ecological and accumulative factors can modulate ontogenetic aging, and that accumulational and ontogenetic mechanisms, though fundamentally independent are yet interwoven. He also suggests that stochastic damage may occur either in a wholly probabilistic fashion (as in somatic DNA damage accumulation), or as the result of genetically-determined programmatic mechanisms, what he refers to as “regular stochastic processes”. Here he has in mind ROS generated by mitochondrial metabolism. A recent case fitting this description is protein aggregation in *C. elegans*, resulting from a programmatic down-regulation of proteostatic mechanisms from around the time of sexual maturation (Labbadia and Morimoto, 2014).

### 7.2 A new multifactorial model based on Dilman’s four models

While Dilman’s ontogenetic theory is a predecessor of the programmatic theory, one can, in a rather recursive fashion, modify and update his four models theory in the light of its newer descendent (Fig. 6B). First, by acknowledging the predominance of the wild-type genotype as the determinant and driver of programmatic mechanisms. This incorporates AP as a major determinative mechanism. Second, to unify ecological and genetic determinants into a single, broad category of causes apart from wild-type function. These represent the major causes of diseases in earlier life, where normal, wild-type function is disrupted. The rearranged scheme emphasizes the centrality of wild-type function, which can be illustrated by a thought experiment. If ecological and genetic factors were, by magic, eliminated, aging would still occur, and there would be little change in either the rate of acceleration of mortality with age (Gompertzian aging) or maximum lifespan. By contrast, if mechanisms of aging from wild-type function were removed, Gompertzian aging would be greatly reduced, though not, one assumes, entirely, due to accumulative mechanisms independent of wild-type function.

The role of inherited mutations in aging in this model requires further explanation. One proposed cause of aging, and mechanism of its evolution, is inherited mutations whose deleterious effects are expressed only later in life, as exemplified by Huntington’s disease (Haldane, 1941; Medawar, 1952). An unexplained feature of this mutation accumulation theory is the mechanism that causes a mutation to remain harmless throughout development and early adulthood, and then become harmful in later life. One possibility is that it is wild-type gene action and programmatic changes that cause the deleterious effects of such mutations to become expressed, or unmasked. Returning to the thought experiment again: according to the unmasking hypothesis, if one eliminated the wild-type gene action component from the revised Dilman model (Fig. 6B), a carrier of the Huntington mutation would not develop the disease.

An important feature of multifactorial models of aging, noted previously (Ezcurra et al., 2018; Gems and de Magalhães, 2021), is their highly context-dependent nature. For a given animal species, the relative contribution of different etiological factors will vary not only between different elements of senescence (e.g. different diseases of aging), but also between individuals. Moreover, the relative contribution of different factors may vary more broadly between species. For example, in humans, environmental, genetic, ontogenetic and accumulative factors all contribute to the development of atherosclerosis, a major determinant of age-related death in humans. The relative importance of these factors will vary between individuals. In one, a major cause may be obesity, and increased cholesterol accumulation; in another, genetic predisposition (e.g. the *APOE4* mutation), in a third, wild-type gene action may act alone. By contrast, the large uterine tumors that are invariably present in senescent *C. elegans* develop due to wholly programmatic mechanisms (Wang et al., 2018a; Wang et al., 2018b), consistent with the view that plastic, programmatic mechanisms play an exceptionally large role in aging in *C. elegans* (Gems et al., 2021; Lohr et al., 2019).

### 7.3 The ontogenesis of the programmatic theory

Scientific discovery is rightly the object of wonder, something to marvel at. In my view, this applies to the programmatic theory. In the early 18th century a fierce priority dispute broke out between Isaac Newton and Gottfried Leibniz arguing over who invented calculus. The modern view is that this was silly, and that both men developed it independently. Such independent, simultaneous discoveries are quite common, and illustrate how scientific innovation is not so much a reflection of individual genius, as interactions of imaginative individuals with a collective and evolving fabric of knowledge. Once the fashion among scientists, these days I-said-it-first-ism risks being interpreted as a character weakness (principally, egotism).

The transpersonal nature of scientific thinking applies well to the programmatic theory, which has evolved over a long period, with certain key contributors. Dilman’s overall scheme, based around his hypothalamic model, was flawed and did not cohere well, yet included a number of prescient elements which contributed to Blagosklonny’s more compelling and better-supported theorem. Dilman himself noted similarities between his ontogenetic theory and Bidder’s hypothesis, formulated in the 1930s (Dilman, 1986). By contrast, de Magalhães’ version of the programmatic theory was developed without knowledge of either Dilman or Blagosklonny’s work. The possibility of the programmatic theory was, as it were, hanging in the air from the early 2000s, and both de Magalhães and Blagosklonny spotted it, with Blagosklonny developing it in more detail with respect to its capacity to explain the origins of disease. Yet the programmatic theory of the mid-2000s forms only part of a wider biology of aging, as discussed here and elsewhere (Gems et al., 2021; Lohr et al., 2019; Maklakov and Chapman, 2019). Of Dilman: he was in many ways ahead of his time and his work deserves greater recognition than it received during his lifetime (at least, outside Russia). He was noted for his creative and integrative thinking style (Napalkov, 2001), characteristics shared by Blagosklonny, and de Magalhães too.

## 8 Concluding remarks

The programmatic theory of aging as developed by Blagosklonny and de Magalhães promises to eventually serve as part of a general framework of understanding aging and the pathophysiology of late-life disease. This could at long last provide the field of biogerontology with an effective explanatory paradigm similar to that provided by the germ theory for the study of infectious disease, and the periodic table for chemistry. More work is needed to explore, test and expand this framework of ideas. This includes elements not discussed here, which will be the subjects of future essays, such as the concept of hypofunction (Maklakov and Chapman, 2019), biological constraint, how quasi-programs are initiated, and how quasi-programs interact in disease pathophysiology.

## Figures and Tables

**Table 1:** Overview of articles by Misha Blagosklonny on theories of aging

Source	Impact	Overview
Conceptual biology: unearthing the gems. Blagosklonny MV, Pardee AB. *Nature* 2002 416 373.	**	Describes conceptual research approach used later to develop aging theories.
Four birds with one stone: RAPA as potential anticancer therapy. Blagosklonny MV, Darzynkiewicz Z. *Cancer Biol. Ther*. 2002 1 359-361.		Initial interest in rapamycin. In normal cells rapamycin blocks angiogenesis and protects against chemotherapeutics; in cancer cells it reduces hypoxia resistance and preferentially inhibits growth where there is an overactive AKT/mTOR pathway.
Cell senescence and hypermitogenic arrest. Blagosklonny MV. *EMBO Rep*. 2003 4 358-362.	**	Proposes that cellular senescence results from exit from the cell cycle combined with hypermitogenic signals (but mTOR not yet mentioned)
Conceptual research and phenomenology-harmonizing slices. Blagosklonny MV. *Cell Cycle* 2003 2 3-4.		Recapitulates *Nature* 2002 416 373.
Aging and immortality: quasi-programmed senescence and its pharmacologic inhibition. Blagosklonny MV. *Cell Cycle* 2006 5 2087-2102.	***	Introduces quasi-program (QP) and hyperfunction (HF) concepts. mTOR HF promotes many diseases of aging. mTOR has AP effects, promoting programs then QPs. This limits lifespan but is only one cause of aging. QPs are a major disease etiology and target of intervention. Rapamycin could prevent senescent multimorbidity.
Cell senescence: hypertrophic arrest beyond the restriction point. Blagosklonny MV. *J. Cell. Physiol*. 2006 209 592-597.		Elaborates cell senescence as hypermitogenic arrest hypothesis, and links to role of mTOR in aging.
An anti-aging drug today: from senescence-promoting genes to anti-aging pill. Blagosklonny MV. *Drug Discov. Today*. 2007 12 218-224.		Recapitulates mTOR-centric model of aging, emphasizing therapeutic potential of rapamycin as anti-aging drug.
Research by retrieving experiments. Blagosklonny MV. *Cell Cycle* 2007 6 1277-1283.	*	A more detailed description of the conceptual research approach, with examples. See *Nature* 2002 416 373.
Program-like aging and mitochondria: instead of random damage by free radicals. Blagosklonny MV. *J. Cell. Biochem*. 2007 102 1389-1399.	*	Enlarged, respiration-defective mitochondria can accumulate in aging cells. mTOR inhibits mitophagy that would otherwise eliminate them.
Paradoxes of aging. Blagosklonny MV. *Cell Cycle* 2007 6 2997-3003.	***	QPs promoted by mTOR/IIS provide a better explanation than DS for life extension by dietary restriction, inhibition of protein synthesis, hormesis and other phenomena. Lifespan is a function of diseases caused by QPs, not a direct consequence of damage accumulation.
Cancer and aging: more puzzles, more promises? Blagosklonny MV, Campisi J. *Cell Cycle* 2008 7 2615-2618.		Discussion of finding from Anisimov lab that metformin can increase lifespan in SHR mice without affecting cancer incidence and onset.
Aging, stem cells, and mammalian target of rapamycin: a prospect of pharmacologic rejuvenation of aging stem cells. Blagosklonny MV. *Rejuvenation Res*. 2008 11 801-808.	**	Proposes the hypothesis that mTOR hyperfunction in stem cells leads to insensitivity to activating stimuli, causing age decline; hence rapamycin should restore stem cell function. More broadly, mTOR promotes signal insensitivity as a brake to growth, and this promotes aging.
Prevention of cancer by inhibiting aging. Blagosklonny MV. *Cancer Biol. Ther*. 2008 7 1520-1524.	*	Summarizes evidence that aging, particularly mTOR activity, promotes cancer, and that mTOR inhibition could prevent aging-related cancer. Aging promotes progression from latent to clinical cancer.
Aging: ROS or TOR. Blagosklonny MV. *Cell Cycle* 2008 7 3344-3354.	***	Better evidence for role of mTOR hyperfunction than ROS as a cause of diseases of aging. Critique of the oxidative damage theory of aging.
Aging-suppressants: cellular senescence (hyperactivation) and its pharmacologic deceleration. Blagosklonny MV. *Cell Cycle* 2009 8 1883-1887.		Discussion of 3 papers from his own lab, in *Cell Cycle* 2009 8. Pharmacological inhibition of TOR, PI-3K, MEK after p16, p21-driven cell cycle arrest inhibits irreversible cellular senescence. Growth pathways drive arrested cells into cellular senescence.
TOR-driven aging: speeding car without brakes. Blagosklonny MV. *Cell Cycle* 2009 8 4055-4059.		Recaps main theory using an analogy. Aging is not like a rusting car, but one without brakes. TOR is the engine, driving pathogenic quasi-programs, rapamycin a brake.
Validation of anti-aging drugs by treating age-related diseases. Blagosklonny MV. *Aging* 2009 1 281-288.	*	A good biomarker of aging to detect anti-aging drug effects is multiple diseases of aging. Anti-aging effects can be detected by retrospective analysis of data from drug trials against individual diseases (data repurposing).
Growth and aging: a common molecular mechanism. Blagosklonny MV, Hall MN. *Aging* 2009 1 357-362.		Concise overview of the biology of mTOR and its role in aging, including a greater emphasis on effects in budding yeast.
Inhibition of S6K by resveratrol: in search of the purpose. Blagosklonny MV. *Aging* 2009 1 511-514.		Response to report in *Aging* that resveratrol inhibits S6 kinase. Resveratrol effects on aging may work through mTOR pathway. Rapamycin action on aging is an unintended effect of a growth inhibiting antifungal; a similar, “side effect” type explanation for resveratrol action on aging is more plausible than the xenohormesis hypothesis.
Linking calorie restriction to longevity through sirtuins and autophagy: any role for TOR. Blagosklonny MV. *Cell Death Dis*. 2010 1 e12.	*	Commentary on Morselli E *et al*. *Cell Death Dis*. 2010 1 e10, which reports that CR and resveratrol increase lifespan by increasing autophagy. Argues against autophagy/lysosome decline as a primary cause of aging.
Calorie restriction: decelerating mTOR-driven aging from cells to organisms (including humans). Blagosklonny MV. *Cell Cycle* 2010 9 683-688.		Recapitulates arguments that DR acts by inhibiting mTOR, and arguments against the molecular damage and allocation hypotheses.
Why human lifespan is rapidly increasing: solving “longevity riddle” with “revealed-slow-aging” hypothesis. Blagosklonny MV. *Aging* 2010 2 177-182.	**	Proposes novel hypothesis for the human mortality rate transition, Vaupel JW. *Nature* 2010 464 536: higher past early mortality of individuals with higher TOR levels.
Rapamycin and quasi-programmed aging: four years later. Blagosklonny MV. *Cell Cycle* 2010 9 1859-1862.	*	Describes how 12 predictions of the key *Cell Cycle* 2006 5 2087 have been borne out by subsequent work.
Why men age faster but reproduce longer than women: mTOR and evolutionary perspectives. Blagosklonny MV. *Aging* 2010 2 265-273.	**	Argues that mTOR activity is higher in men, and reiterates Dilman’s theory that menopause is due to a quasi-program: run-on of the decline in hypothalamic sensitivity to inhibition by estrogen that triggers puberty by allowing FSH production. Later hypothalamic insensitivity leads to FSH over-production, causing a futile acceleration of follicular atresia.
Revisiting the antagonistic pleiotropy theory of aging: TOR-driven program and quasi-program. Blagosklonny MV. *Cell Cycle* 2010 9 3151-3156.	**	Critique of Kirkwood TB. *Cell* 2005 120 437 claim that p53 exhibits AP. Argues instead that mTOR and IIS pathway genes exhibit AP, and rapamycin is a reversed-AP drug.
Increasing healthy lifespan by suppressing aging in our lifetime: preliminary proposal. Blagosklonny MV. *Cell Cycle* 2010 9 4788-4794.		Introduces term *post-aging syndrome* for senescence where mTOR hyperfunction is suppressed.
Why the disposable soma theory cannot explain why women live longer and why we age. Blagosklonny MV. *Aging* 2010 2 884-887.	*	Critique of Kirkwood T. “Why women live longer.” *Sci Am* 2010 303 34, and disposable soma. Recapitulates arguments from *Aging* 2010 2 265.
Metformin and sex: Why suppression of aging may be harmful to young male mice. Blagosklonny MV. *Aging* 2010 2 897-9.		Commentary on finding that metformin increases mortality rate in young adult male mice. Elaboration of “revealed slow aging” hypothesis (*Aging* 2010 2 177).
Cell cycle arrest is not senescence. Blagosklonny MV. *Aging* 2011 3 94-101.	**	Development and extension of the hypermitogenic arrest idea, including *physiological senescence*: growth promotion in quiescent, wild-type cells in vivo leads to hyperfunctional state; view of cancer cells as *pro-senescent*: pathogenic hyperfunction seen in senescent cells also occurs in cancer cells, contributing to hallmarks of cancer.
Progeria, rapamycin and normal aging: recent breakthrough. Blagosklonny MV. *Aging* 2011 3 685-691.		Commentary on finding that rapamycin suppresses pro-senescent phenotype in cells from Hutchinson Gilford progeria patients (*Sci. Transl. Med*. 2011 3 89ra58). mTOR promotes progeria, including life-shortening effects of obesity. Rapamycin could be a treatment for progeria and normal aging.
Hormesis does not make sense except in the light of TOR-driven aging. Blagosklonny MV. *Aging* 2011 3 1051-1062.	**	This argues against the interpretation that hormetic effects on aging reflect suppression of molecular damage. Proposes two types of hormesis. *Hormesis A* acts by inhibiting mTOR. *Hormesis B* induces aging tolerance: resistance to catastrophic complications of aging (e.g. ischemic conditioning).
Molecular damage in cancer: an argument for mTOR-driven aging. Blagosklonny MV. *Aging* 2011 3 1130-1141.	**	Molecular damage (mutation) is important in cancer, but this is different to the standard damage/wear-and-tear view of aging. Suggests an almost developmental view of cancer ontogeny: “numerous random mutations are selected for non-random activation of mTOR”.
NCI’s provocative questions on cancer: some answers to ignite discussion. Blagosklonny MV. *Oncotarget* 2011 2 1352-1367.	*	Response to US National Cancer Institute’s 24 questions about cancer. Includes good account of links between aging, obesity and cancer (particularly those involving mTOR).
Cell cycle arrest is not yet senescence, which is not just cell cycle arrest: terminology for TOR-driven aging. Blagosklonny MV. *Aging* 2012 4 159-165.	**	Develops concept of hypermitogenic arrest, using new terminology including *gerogenic conversion/ geroconversion*, and *gerogenic oncogenes/gerogenes*, to give a more conceptually integrated account of the roles of PI3K/RAS/mTOR action in aging and cancer.
Once again on rapamycin-induced insulin resistance and longevity: despite of or owing to. Blagosklonny MV. *Aging* 2012 4 350-358.		Response to study of diabetes-like condition in mice under chronic rapamycin treatment (*Science* 2012 335 1638). Argues this this “starvation diabetes”, a form of benevolent diabetes, and not an arguments against rapamycin safety.
Prospective treatment of age-related diseases by slowing down aging. Blagosklonny MV. *Am. J. Pathol*. 2012 181 1142-1146.		Reiteration of preventative approach to reduce age-related disease using rapamycin. Describes how multiple forms of hyperfunction contribute to atherosclerosis.
How to save Medicare: the anti-aging remedy. Blagosklonny MV. *Aging* 2012 4 547-552.		Deceleration of aging would reduce healthcare costs, and could be achieved by mTOR inhibitors (e.g. rapamycin).
Rapalogs in cancer prevention: anti-aging or anticancer? Blagosklonny MV. *Cancer Biol. Ther*. 2012 13 1349-1354.		Short review of evidence that rapalogs can prevent cancer, at least in part by slowing aging.
Answering the ultimate question “what is the proximal cause of aging?” Blagosklonny MV. *Aging* 2012 4 861-877.	*	Response to Zimniak P. “What is the proximal cause of aging?” *Front. Genet*. 2012 3 189, a critique of the hyperfunction theory. Reiterates the theory, with emphasis on disease causation.
Common drugs and treatments for cancer and age-related diseases: revitalizing answers to NCI’s provocative questions. Blagosklonny MV. *Oncotarget* 2012 3 1711-1724.		Follow-up to *Oncotarget* 2011 2 1352. More on aging, obesity and cancer. A number of existing drugs (e.g. ACE inhibitors) may have cancer preventative effects by inhibiting mTOR.
Hypoxia, MTOR and autophagy: converging on senescence or quiescence. Blagosklonny MV. *Autophagy* 2013 9 260-262.		Discussion of his own paper, *PNAS* 2012 109 13314. Hypoxia promotes geroconversion by inhibiting mTOR. Also discusses possible significance of mTOR-autophagy spatial coupling compartment (TASCC) in senescent cells.
Big mice die young but large animals live longer. Blagosklonny MV. *Aging* 2013 5 227-233.	**	Presents hypothesis that selection for slower aging results in slower development. Large body size (within species) can result from growing faster (leading to faster aging) or (between species) developing longer (leading to slower aging).
MTOR-driven quasi-programmed aging as a disposable soma theory: blind watchmaker vs. intelligent designer. Blagosklonny MV. *Cell Cycle* 2013 12 1842-1847.		Recapitulates arguments for hyperfunction theory and against disposable soma theory. Hyperfunction theory is disposable soma theory 2. Aging is quasi-programmed by the blind watchmaker (c.f. Dawkins).
M(o)TOR of aging: MTOR as a universal molecular hypothalamus. Blagosklonny MV. *Aging* 2013 5 490-494.	*	Reaction to *Nature* 2013 497 211, evidence that inflammation in the hypothalamus promotes mouse aging. Argues that mTOR acts as a molecular hypothalamus in the cell, integrating signals generated by insulin, mitogens, cytokines, oxygen, and nutrients.
Rapamycin extends life- and health span because it slows aging. Blagosklonny MV. *Aging* 2013 5 592-598.		Cogent critique of *J Clin Invest*. 2013 123 3272 study arguing that rapamycin prevents disease but not aging. Reiterates key points made previously.
Damage-induced aging and perpetual motion. Blagosklonny MV. *Cell Cycle* 2013 12 2709-2710.		US Patent Office now refuses to grant patents for perpetual motion machines. Should a similar policy be made for papers claiming to show that aging is caused by molecular damage? Reiterates key points made previously.
Aging is not programmed: genetic pseudo-program is a shadow of developmental growth. Blagosklonny MV. *Cell Cycle*. 2013 12 3736-3742.		Account of hyperfunction theory aiming to correct the misconception that the description of aging as quasi-programmed means that the hyperfunction theory is a programmed aging theory.
TOR-centric view on insulin resistance and diabetic complications: perspective for endocrinologists and gerontologists. Blagosklonny MV. *Cell Death Dis*. 2013 4 e964.	**	Account of how pathophysiology of type 2 diabetes, including retinopathy, nephropathy and neuropathy, can be understood as resulting from mTOR hyperfunction rather than hyperglycemia.
Selective anti-cancer agents as anti-aging drugs. Blagosklonny MV. *Cancer Biol. Ther*. 2013 14 1092-1097.		Surveys anticancer drugs that target proteins that also promote aging. Using them geroprotectively will protect against cancer. Some reiteration of *Cancer Biol. Ther*. 2012 13 1349.
Koschei the immortal and anti-aging drugs. Blagosklonny MV. *Cell Death Dis*. 2014 5 e1552.		Review of rapamycin protection against various senescent pathologies (including in humans), and also obesity. High rapamycin mimics starvation-induced pseudo-diabetes (benign, reversible glucose intolerance). Effects of rapamycin could be enhanced by exercise, low calorie diet and combination with other drugs (e.g. metformin, aspirin, angiotensin II receptor blockers).
Geroconversion: irreversible step to cellular senescence. Blagosklonny MV. *Cell Cycle* 2014 13 3628-3635.		Reiterates the two stage (quiescence, geroconversion) model of cell senescence, emphasizing the importance of the latter in disease etiology.
Rejuvenating immunity: “anti-aging drug today” eight years later. Blagosklonny MV. *Oncotarget* 2015 6 19405-19412.		Response to *Sci Transl Med*. 2014 6 268ra179 showing that Everolimus improves immune function in the elderly. Reiterates mTOR-centric model, and the potential of mTOR inhibitors to retard aging.
From rapalogs to anti-aging formula. Blagosklonny MV. *Oncotarget* 2017 8 35492-35507.		Updates Koschei formula paper (2014), discussing how anti-aging drugs could be combined. Accumulating evidence of effects against multiple senescent pathologies of e.g. angiotensin II inhibitors, aspirin, PDE5 inhibitors.
Does rapamycin slow down time? Blagosklonny MV. *Oncotarget* 2018 9 30210-30212.	*	Short review viewing rate of developmental processes in senescence as biological time, and noting effects of mTOR/rapamycin on biological clocks (circadian, epigenetic).
Disease or not, aging is easily treatable. Blagosklonny MV. *Aging* 2018 10 3067-3078.	*	Account of aging as clinical diseases, their pre-diseases, and pre-pre-diseases in the primary causes of aging (hyperfunction).
Rapamycin, proliferation and geroconversion to senescence. Blagosklonny MV. *Cell Cycle* 2018 17 2655-2665.		Updated reiteration of the two stage (quiescence, geroconversion) model of cell senescence, including discussion of the problem of defining cellular senescence in vivo.
Paradoxes of senolytics. Blagosklonny MV. *Aging* 2018 10 4289-4293.	*	Senolytic drugs counteract hyperfunction not damage. Discussion of absolute vs relative hyperfunction.
The mystery of the ketogenic diet: benevolent pseudo-diabetes. Blagosklonny MV. *Cell Cycle* 2019 18 2157-2163.		Like rapamycin, ketogenic diets (KDs) extend mouse lifespan and induce pseudo-diabetes. KDs (e.g. Atkins diet) have promise as treatments for human aging.
Fasting and rapamycin: diabetes versus benevolent glucose intolerance. Blagosklonny MV. *Cell Death Dis*. 2019 10 607.		Updated reiteration of how high rapamycin mimics starvation-induced pseudo-diabetes (benign, reversible glucose intolerance).
Rapamycin for longevity: opinion article. Blagosklonny MV. *Aging* 2019 11 8048-8067.	*	Updated recapitulation of potential of rapamycin as an anti-aging drug, including critique of arguments against this.
Rapamycin for the aging skin. Blagosklonny MV. *Aging* 2019 11 12822-12826.	*	Response to *Geroscience* 2019 41 861 study showing that topical rapamycin ameliorates skin senescence.
From causes of aging to death from COVID-19. Blagosklonny MV. *Aging* 2020 12 10004-10021.		Quasi-programmed hyperfunction, promoted by mTOR, contributes to inflammaging, leading to lethal cytokine storms and increased death rates in older COVID-19 patients.
The goal of geroscience is life extension. Blagosklonny MV. *Oncotarget* 2021 12 131-144.		Critique of the use of increased healthspan rather than increased lifespan as criterion for identifying anti-aging drugs using animal models. Reviews effects of diverse drugs on lifespan in animal models.
DNA- and telomere-damage does not limit lifespan: evidence from rapamycin. Blagosklonny MV. *Aging* 2021 13 3167-3175.	*	Recent studies show that rapamycin does not increase lifespan in DNA repair- and telomerase-deficient mice. Argues that this is evidence that DNA damage is not life limiting in normal aging. Good example of use of the onion model (see glossary).
Response to the thought-provoking critique of hyperfunction theory by Aubrey de Grey. Blagosklonny MV. *Rejuvenation Res*. 2021 24 170-172.		Rebuttal to various objections defending the damage maintenance paradigm, similar in character to those made by P. Zimniak. See *Aging* 2012 4 861.
No limit to maximal lifespan in humans: how to beat a 122-year-old record. Blagosklonny MV. *Oncoscience* 2021 8 110-119.		Compression of morbidity, sometimes stated as a goal of research on aging, is unrealistic. Apparent compression of morbidity in centenarians is due to insufficient medical care. Anti-aging treatments cause *relative* compression of morbidity only.
The hyperfunction theory of aging: three common misconceptions. Blagosklonny MV. *Oncoscience* 2021 8 103-107.		Hyperfunction can be absolute (increased activity) or relative. The theory does not argue against a role of molecular damage in aging. The theory originates from the cellular model of geroconversion.
Anti-aging: senolytics or gerostatics (unconventional view). Blagosklonny MV. *Oncotarget* 2021 12 1821-1835.	**	Critical reassessment of senotherapy. Many cells in vivo undergo geroconversion and become gerogenic without becoming fully senescent. Is reduction in senescent cell number after senotherapy actually due to senescent cell death? Could increases in mouse lifespan from treatment with dasatinib and quercetin be due to mTOR inhibition?

Stars indicate this author’s estimation of impact in terms of aging theory.

*** key source presenting major new ideas.

** presents notable new concepts or perspectives.

* notable minor additions to the ideas framework. Where ideas are presented in several different essays, the first presentation is usually estimated as of higher impact.

## Glossary

Adaptive cellular senescence: A type of programmed cellular senescence, e.g. where “senescent” fibroblasts contribute to wound healing (Demaria et al., 2014). This contrasts with quasi-programmed, non-adaptive cellular senescence. In some cases cellular senescence may have both adaptive and futile elements, as when a cell leaves the cell cycle due to DNA damage (programmed, adaptive) and then becomes hypertrophic and hypersecretory (quasi-programmed, non-adaptive).
Antagonistic pleiotropy (AP): Where action of a given gene is both beneficial and detrimental to fitness. If the latter occurs later in life and is therefore subject to weaker selection, such a gene may be favored by natural selection, and promote aging (Williams, 1957).
Damage/maintenance paradigm: Theory that aging is largely caused by accumulation of molecular damage, which can be prevented by somatic maintenance functions. Various theories of aging are based on this broad assumption.
Disposable soma: Theory proposing that natural selection favors investment of limited resources into reproduction rather than somatic maintenance, accelerating damage accumulation and, therefore, senescence (Kirkwood, 1977).
Hyperfunction: Where wild-type gene action and biological function actively leads to senescent pathology, as opposed to passive random damage or wear and tear (Blagosklonny, 2006a).
Onion model (new term): This views aging as a nested series of life-limiting pathologies (Blagosklonny, 2006a; de Magalhães, 2012). Thus, lifespan is a function not of the overall aging process, but of those processes generating life-limiting pathologies. Which pathologies are life-limiting depends upon environment and animal species.
Programmed aging: Senescence caused by a relatively ordered series of biological processes that promotes fitness via inclusive fitness or group fitness. Evolutionary theory predicts that this does not occur in most animal species, but can in some cases (Galimov and Gems, 2021; Lohr et al., 2019).
Programmatic aging: Where senescence is genetically determined, including where complex biological processes contribute to senescence, but not necessarily to fitness (cf. quasi-programs, costly programs).
Quasi-programmed aging: Senescence caused by a relatively ordered series of biological processes that does not promote fitness; may occur due to futile run-on of wild-type programs that promote fitness earlier in life (Blagosklonny, 2006a).
Run-on: Futile continuation of gene function or processes in later life, leading to pathology (de la Guardia et al., 2016) (cf. quasi-program).
Selection shadow: Decrease in selection with increasing age, leading to weaker selection against genes with deleterious effects on fitness and health the later in life those effects are expressed. Environmental factors that increase mortality rate (e.g. predators, infectious pathogens, starvation) can deepen the selection shadow.
Senescence: The overall process of deterioration with age or the resulting pathological condition (not to be confused with *cellular senescence* (*sensu* Hayflick), which is a particular form of cell cycle arrest affecting some vertebrate cell types). Although *aging* has several meanings, in the biological context it is usually synonymous with senescence.
Ultimate-proximate theories of aging: These combine explanations of the evolutionary (ultimate) and mechanistic (proximate) causes of aging into a single integrated account.
